# On the reduction of imaging time-points for dosimetry in radionuclide therapy

**DOI:** 10.1186/s40658-025-00721-y

**Published:** 2025-02-06

**Authors:** Johan Gustafsson, Jan Taprogge

**Affiliations:** 1 Medical Radiation Physics, Lund, Lund University, Lund, Sweden; 2 Joint Department of Physics, Royal Marsden NHSFT, Sutton, UK; 3 The Institute of Cancer Research, London, UK

**Keywords:** Radionuclide therapy, Dosimetry, Uncertainty, Curve fitting

## Abstract

**Background:**

The aim was to develop a theoretical framework for how errors in estimated activities propagate to a dispersion in time-integrated activity in radionuclide-therapy dosimetry and how this affects the comparison of radionuclide-therapy dosimetry schemes.

**Methods:**

Formulae for the variance of relative errors of estimated time-integrated activities and relative differences in time-integrated activities between measurement schemes when one or more time-points are removed were derived using the law of propagation of uncertainty for a population of time-activity-curve parameters. The formulae were derived under the assumptions of fixed coefficients of variation for estimated activities, and underlying mono-exponential curves. Analytical predictions were compared with results from numerical simulations and data for kidneys, liver, and spleen from a data-set of 18 patients treated with ^177^Lu-DOTA-TATE.

**Results:**

The dispersion in time-integrated activity is minimized if the time-points used for curve fitting have a large dispersion and are centered over the mean of $\tau ={\lambda }_{\text{eff}}^{-1}$ over the population, where ${\lambda }_{\text{eff}}$ is the effective decay constant (*i.e.*, the sum of the biological and physical decay constants). For large dispersions of decay constants in the population, the centering of time-points becomes gradually less important. The analytical expressions replicated the main trends from the numerical simulations. Furthermore, the analytical expressions predicted correctly the optimal reduced imaging schedule in 9 of 12 pairwise comparisons between schedules for patients.

**Conclusions:**

The dispersion of errors and deviations in estimated time-activity curves can be predicted using simple formulae. These formulae have the potential to be used for optimization of dosimetry measurement schemes for established and new radiopharmaceuticals as long as the mean and dispersion of biological half-lives are known in the patient population.

**Supplementary Information:**

The online version contains supplementary material available at 10.1186/s40658-025-00721-y.

## Background

Patient-specific dosimetry, *i.e.*, estimation of absorbed doses to organs and tumours, is a potential tool for optimization and individualization of radionuclide therapy (RNT) (1, 2). As part of the transition of dosimetry-based RNT from a research setting to clinical routine, a number of papers have studied the possibility to simplify dosimetry measurement schemes while retaining a sufficient accuracy of absorbed-dose estimates in relation to the clinical requirements (3–10), for example by reducing the number of time-points at which images are acquired. The reduction of the number of imaging time-points has advantages in terms of simplified logistics and is thus of considerable interest for bringing dosimetry-based RNT to clinical use. However, there is a concern that a reduced number of measurement time-points may deteriorate the accuracy of resulting absorbed-dose estimates (11) which could ultimately lead to suboptimal treatment or overexposure of organs-at-risk.

The basis for RNT dosimetry is often serial quantitative imaging for the estimation of activity or activity concentration in organs and tumours. From these estimates at a discrete number of time-points, a time-activity curve (TAC) is fitted and integrated over time. Each individual estimated activity is associated with an uncertainty. These uncertainties will combine and propagate into an uncertainty in time-integrated activity (TIA), and by extension an uncertainty in absorbed dose. Since each activity measurement is relatively expensive in terms of patient time and hospital resources, only a small number of measurements are typically available to which mono-, bi-, or multi-exponential functions are fitted (12, 13). The small number of measurements available makes the placement of time-points critical to achieve an as favourable propagation of errors as possible.

One of the most common strategies for studying the simplification of dosimetry measurement schemes has been to start from a larger number of measurements and then compare the results from a reduced number of measurements to those obtained from the full dataset (3–6, 8, 14). Other studies have used a simulation approach. For example, Rinscheid et al. (15) and Rinscheid et al. (16) studied precision and bias for different sampling schedules starting from TACs from a pharmacokinetic model of ^177^Lu-PSMA. Typically, simplified methods are found to be feasible in the sense that the number of measurements can be reduced from 3 to 2, say, with only a small effect on TIA given that the removed time-point is chosen appropriately. The need for late imaging time-points has been emphasized in relation to the effective half-life of the slowest phase of the pharmaceutical (17, 18).

To complement the extensive body of empirical data on the simplification and optimization of RNT dosimetry measurement schemes, we believe a better theoretical understanding of the problem would be beneficial. A theoretical framework would clarify the results obtained in different studies and guide future research or the development of optimal imaging schedules for new radiopharmaceuticals or applications. A general framework for propagating uncertainty through a calculation is given by the guide to the expression of uncertainty in measurement (GUM) (19) summarized in the law of propagation of uncertainty (LPU), generalized to the case of multiple separate quantities in GUM supplement 2 (20). The GUM framework is based on a linearization approximation, which allows for analytic propagation of the covariance matrix through the calculation process provided that the uncertainties are reasonably low, and the function is well described by a linear approximation. Even if derived relationships will by necessity be limited by the simplifying assumptions and approximations involved, the ability to derive closed expressions for the uncertainty has the potential to provide an intuitive understanding of the problem that empirical data or simulation studies cannot give. For example, Flux et al. (21) used such uncertainty propagation for understanding the effects of using different isotopes for pre-therapeutic dosimetry and therapy, demonstrating how uncertainty in estimates are increased when the physical half-life of the radionuclide used in the pre-therapeutic session is shorter than the one used for therapy.

The aim of this paper is to derive analytical expressions for the uncertainty propagation through the TAC fitting and integration process under the assumption of fixed coefficient of variation (CV) in estimated activity, for the purpose of comparing dosimetry schemes with different number of time-points with respect to the impact of random errors in estimated activity concentration on TIA. Derived formulae are verified with numerical simulations and their use demonstrated for patient data.

## Theory

The theory on which this paper is based will be discussed in terms of the estimation of TIA. However, the theory is general and can be applied also to the fitting and integration of other quantities used in RNT dosimetry, *e.g.*, activity concentration (22) or absorbed-dose rate. A summary of the variables used in the derivation is given in Table 1.

**Table 1:** Summary of variables used in the development of the theory

Variable	Meaning	Variable	Meaning
$\widetilde{A}\left(\mathbf{p}\right)$	TIA for a TAC with pharmacokinetic parameters $\mathbf{p}$	$\mathbf{0}$	Zero-matrix
$A(t,\mathbf{p})$	TAC with pharmacokinetic parameters $\mathbf{p}$ and time $t$	$\mathbf{p}\mathbf{^{\prime}}$	Column matrix with estimated TAC parameters from $\mathbf{A}^{\prime}$
$\mathbf{p}$	Column matrix of pharmacokinetic parameters	$\mathbf{p}\mathbf{^{\prime}}\mathbf{^{\prime}}$	Column matrix with estimated TAC parameters from $\mathbf{A}^{\prime\prime}$
$t$	Time post injection	$\mathbf{J}\boldsymbol{^{\prime}}$	Jacobian for the least-squares fit to $\mathbf{A}^{\prime}$
${A}_{i}$	Estimated activity at time ${t}_{i}$	$\mathbf{J}\boldsymbol{^{\prime}}\boldsymbol{^{\prime}}$	Jacobian for the least-squares fit to ${\mathbf{A}}^{\prime\prime}$
$\mathbf{A}^{\prime}$	Column matrix with estimated activities ${A}_{1}$ to ${A}_{n}$	$\mathbf{W}$	Block-diagonal matrix of ${\mathbf{W}}^{\prime}$ and ${\mathbf{W}}^{\prime\prime}$
${{\varvec{\Sigma}}}_{{\mathbf{A}}^{\boldsymbol{^{\prime}}}}$	Covariance matrix for $\mathbf{A}^{\prime}$	$\mathbf{J}$	Block-diagonal matrix of $\mathbf{J}\boldsymbol{^{\prime}}$ and $\mathbf{J}\boldsymbol{^{\prime}}\boldsymbol{^{\prime}}$
$E\left[\cdot \right]$	Expectation operator	$${{\varvec{\Sigma}}}_{\left[\begin{array}{c}{\mathbf{p}}{\prime}\\ {\mathbf{p}}^{{\prime}{\prime}}\end{array}\right]}$$	Covariance matrix of $\mathbf{p}\mathbf{^{\prime}}$ and $\mathbf{p}\mathbf{^{\prime}}\mathbf{^{\prime}}$ combined
$r$	CV for estimated activities	${\varvec{\Upsilon}}$	Jacobian matrix for the calculation of TIA from TAC parameters
${\widetilde{A}}_{1}$	TIA estimated from $\mathbf{A}^{\prime}$	$\mathbf{U}$	Block-diagonal matrix with diagonal elements ${\varvec{\Upsilon}}$
$E\left[\cdot | \mathbf{p}\right]$	Conditional expectation under $\mathbf{p}$	$${{\varvec{\Sigma}}}_{\left[\begin{array}{c}{\widetilde{A}}_{1}\\ {\widetilde{A}}_{2}\end{array}\right]}$$	Covariance matrix for ${\widetilde{A}}_{1}$ and ${\widetilde{A}}_{2}$
$\varepsilon$	Relative error ${\widetilde{A}}_{1}/\widetilde{A}\left(\mathbf{p}\right)-1$	$A\left(t\right)$	Activity as function of time
$V\left[\cdot \right]$	Variance operator	${A}_{0}$	Activity at time $t=0$
$V\left[\cdot | \mathbf{p}\right]$	Conditional variance under $\mathbf{p}$	${\lambda }_{\text{phys}}$	Physical-decay constant
${\varvec{A}}^{\prime\prime}$	Column matrix containing the $n-k$ first elements in $\mathbf{A}^{\prime}$	${\lambda }_{\text{biol}}$	Biological-decay constant
${\widetilde{A}}_{2}$	TIA estimated from ${\varvec{A}}^{\prime\prime}$	$\overline{{t }^{\prime}}$	Average of time-points in $\mathbf{A}^{\prime}$
$\mathbf{A}$	Concatenated column matrix of $\mathbf{A}^{\prime}$ and ${\varvec{A}}^{\prime\prime}$	${s^{\prime}}^{2}$	Variance of time-points in $\mathbf{A}^{\prime}$
${{\varvec{\Sigma}}}_{\mathbf{A}}$	Covariance matrix of $\mathbf{A}$	$\overline{{t }^{{\prime}{\prime}}}$	Average of time-points in $\mathbf{A}^{\prime\prime}$
${\mathbf{W}}^{\prime}$	Weighting matrix for the TAC fit to $\mathbf{A}^{\prime}$	${{s}^{{\prime}{\prime}}}^{2}$	Variance of time-points in $\mathbf{A}^{\prime\prime}$
${\mathbf{W}}^{{\prime}{\prime}}$	Weighting matrix for the TAC fit to ${\varvec{A}}^{\prime\prime}$	$\tau$	The inverse of the effective-decay constant

### General framework

Consider a TIA $\widetilde{A}\left(\mathbf{p}\right)$ for a TAC $A\left(t,\mathbf{p}\right)$ parametrized by a vector of parameters $\mathbf{p}$ and time $t$. The TIA is estimated from a set of activity measurements $\mathbf{A}^{\prime}={\left[{A}_{1}, {A}_{2},\dots {A}_{n}\right]}^{\text{T}}$ measured at time ${t}_{1}$, ${t}_{2}$,…, ${t}_{n}$, where $n$ is larger than or equal to the number of elements in $\mathbf{p}$, by least-squares fitting. The measurement of each activity will, in general, be associated with systematic errors, common for all data-points, and random errors unique to every point. With respect to curve fitting, the relevant variability in estimated activities is associated with random errors, and systematic errors will only serve to scale the estimated TAC and not be diminished by averaging effect of curve-fitting and integration. Hence, systematic errors will be ignored in the current theoretical treatment which focuses mainly on the comparison of different measurement schedules and not the absolute error in the TIA compared to the underlying truth. For a fixed geometry, the variability associated with the random component can reasonably be assumed to be proportional to the expected value and independent (11, 21), assuming that, for a given geometry, the main cause of variation is caused by processes such as segmentation of reconstructed images and partial-volume correction (23, 24) rather than from the random nature of photon emission and detection. That is, the covariance matrix for the estimated activities is

1 $$\begin{array}{*{20}c} {{{\varvec{\Sigma}}}_{{{\mathbf{A^{\prime}}}}} = r^{2} \left[ {\begin{array}{*{20}c} {A^{2} \left( {t_{1} } \right)} & 0 & \cdots & 0 \\ 0 & {A^{2} \left( {t_{2} } \right)} & \cdots & 0 \\ \vdots & \vdots & \ddots & \vdots \\ 0 & 0 & \cdots & {A^{2} \left( {t_{n} } \right)} \\ \end{array} } \right]} \\ \end{array}$$

where $r$ is the CV of estimated activities.

Let  ${\widetilde{A}}_{1}$ be an estimate of the of the time integrated activity from $\mathbf{A}\mathbf{^{\prime}}$. Even if not necessarily strictly true, we will assume the estimate to be unbiased, *i.e.*, $E\left[{\widetilde{A}}_{1}| \mathbf{p}\right]=\widetilde{A}\left(\mathbf{p}\right)$. For a single estimate, the relative error is

2 $$\begin{array}{*{20}c} {\varepsilon = \frac{{\tilde{A}_{1} }}{{E\left[ {\tilde{A}_{1} | {\mathbf{p}}} \right]}} - 1 = \frac{{\tilde{A}_{1} }}{{\tilde{A}\left( {\mathbf{p}} \right)}} - 1} \\ \end{array}$$

A measure of the precision of the estimate becomes

3 $$\begin{array}{*{20}c} {V\left[ \varepsilon \right] = E\left[ {V\left( {\varepsilon |{\mathbf{p}}} \right)} \right] + V\left[ {E\left( {\varepsilon |{\mathbf{p}}} \right)} \right] = E\left[ {V\left( {\varepsilon |{\mathbf{p}}} \right)} \right]} \\ \end{array}$$

where the law of total variance has been applied and the assumption of an unbiased estimator, leading to $E\left[\varepsilon |\mathbf{p}\right]=0 \forall \mathbf{p}$, has been used.

For patient studies, the typical mode-of-operation for investigating different RNT dosimetry imaging schedules is to compare the time-integrated activities estimated with full datasets with those where one or more measurements have been removed. For such a scenario, let ${\widetilde{A}}_{2}$ be an estimate of the TIA that only uses $n-k$ of the data points in $\mathbf{A}^{\prime}$, with $k$ small enough so that $n-k$ is larger than the number of parameters in $\mathbf{p}$. Since there is no requirement for the time-points to be listed in chronological order, we can assume these to be the ${\varvec{A}}^{\prime\prime}={\left[{A}_{1}, {A}_{2},\dots {A}_{n-k}\right]}^{\text{T}}$ at ${t}_{1}$, ${t}_{2}$,…, ${t}_{n-k}$ without loss of generality. The relative difference between the two estimates is

4 $$\begin{array}{*{20}c} {\delta = \frac{{\tilde{A}_{2} }}{{\tilde{A}_{1} { }}} - 1} \\ \end{array}$$

The variation in relative differences is characterized by

5 $$\begin{array}{*{20}c} {V\left[ \delta \right] = E\left[ {V\left( {\delta |{\mathbf{p}}} \right)} \right] + V\left[ {E\left( {\delta |{\mathbf{p}}} \right)} \right] \approx E\left[ {V\left( {\delta |{\mathbf{p}}} \right)} \right]} \\ \end{array}$$

where, again the property of unbiased estimators has been used, leading to $E\left[\delta |\mathbf{p}\right]\approx 0 \forall \mathbf{p}$. The conditional variance evaluates to

6 $$\begin{array}{*{20}c} {V\left[ {\delta |{\mathbf{p}}} \right] = \frac{1}{{\tilde{A}^{2} \left( {\mathbf{p}} \right)}}\left( {V\left[ {\tilde{A}_{1} |{\mathbf{p}}} \right] + V\left[ {\tilde{A}_{2} |{\mathbf{p}}} \right] - 2{\text{cov}}\left[ {\tilde{A}_{1} , \tilde{A}_{2} |{\mathbf{p}}} \right]} \right)} \\ \end{array}$$

using the LPU.

### Covariance between estimates

To calculate $V\left[\varepsilon \right]$ and $V\left[\delta \right]$, the conditional variances of  ${\widetilde{A}}_{1}$ and ${\widetilde{A}}_{2}$ are required as well as the conditional covariance between them. To obtain the covariance in addition to the individual variances, the fitting of time-activity curves to the full and reduced datasets need to be considered in combination. For this purpose, define the column matrix

7 $$\begin{array}{*{20}c} {A = \left[ {\begin{array}{*{20}c} {{\mathbf{A^{\prime}}}} \\ {{\mathbf{A^{\prime\prime}}}} \\ \end{array} } \right]} \\ \end{array}$$

for which the covariance matrix is

8 $$\begin{array}{*{20}c} {{{\varvec{\Sigma}}}_{{\mathbf{A}}} = r^{2} \left[ {\begin{array}{*{20}c} {{\mathbf{W^{\prime}}}^{ - 1} } & {\left[ {\begin{array}{*{20}c} {{\mathbf{W^{\prime\prime}}}^{ - 1} } \\ \mathbf{0} \\ \end{array} } \right]} \\ {\left[ {\begin{array}{*{20}c} {{\mathbf{W^{\prime\prime}}}^{ - 1} } & \mathbf{0} \\ \end{array} } \right]} & {{\mathbf{W^{\prime\prime}}}^{ - 1} } \\ \end{array} } \right]} \\ \end{array}$$

where

9 $$\begin{array}{*{20}c} {{\mathbf{W^{\prime}}} = \left[ {\begin{array}{*{20}c} {A^{ - 2} \left( {t_{1} } \right)} & 0 & \cdots & 0 \\ 0 & {A^{ - 2} \left( {t_{2} } \right)} & \cdots & 0 \\ \vdots & \vdots & \ddots & \vdots \\ 0 & 0 & \cdots & {A^{ - 2} \left( {t_{n} } \right)} \\ \end{array} } \right]} \\ \end{array}$$

10 $$\begin{array}{*{20}c} {{\mathbf{W^{\prime\prime}}} = \left[ {\begin{array}{*{20}c} {A^{ - 2} \left( {t_{1} } \right)} & 0 & \cdots & 0 \\ 0 & {A^{ - 2} \left( {t_{2} } \right)} & \cdots & 0 \\ \vdots & \vdots & \ddots & \vdots \\ 0 & 0 & \cdots & {A^{ - 2} \left( {t_{n - k} } \right)} \\ \end{array} } \right]} \\ \end{array}$$

and $\mathbf{0}$ denotes a zero-matrix of the appropriate size. The non-zero covariances in Eq. 8 result from that the activity estimates common to $\mathbf{A}\mathbf{^{\prime}}$ and $\mathbf{A}^{\prime\prime}$ are identical and hence perfectly correlated.

Let $\mathbf{p}\mathbf{^{\prime}}$ and $\mathbf{p}\mathbf{^{\prime}}\mathbf{^{\prime}}$ be the estimates of $\mathbf{p}$ associated with estimates ${\widetilde{A}}_{1}$ and ${\widetilde{A}}_{2}$, and $\mathbf{J}\boldsymbol{^{\prime}}$ and $\mathbf{J}\boldsymbol{^{\prime}}\boldsymbol{^{\prime}}$ the Jacobians for each such non-linear least-squares fit with weighting matrices $\mathbf{W}^{\prime}$ and $\mathbf{W}^{\prime\prime}$. Under a linearization approximation, the covariance matrix of ${\left[{\mathbf{P}}^{{\prime}\text{T}} {\mathbf{P}}^{{\prime}{\prime}\text{T}}\right]}^{\text{T}}$ becomes (Appendix 1)

11 $$\begin{array}{*{20}c} {{{\varvec{\Sigma}}}_{{\left[ {\begin{array}{*{20}c} {{\mathbf{p^{\prime}}}} \\ {{\mathbf{p^{\prime\prime}}}} \\ \end{array} } \right]}} = \left( {{\mathbf{J}}^{{\text{T}}} {\mathbf{WJ}}} \right)^{ - 1} {\mathbf{J}}^{{\text{T}}} \mathbf{W}{{\varvec{\Sigma}}}_{{\mathbf{A}}} \mathbf{WJ}\left( {{\mathbf{J}}^{{\text{T}}} {\mathbf{WJ}}} \right)^{ - 1} } \\ \end{array}$$

where

12 $$\begin{array}{*{20}c} {W = \left[ {\begin{array}{*{20}c} {{\mathbf{W}^{\prime}}} & \mathbf{0} \\ \mathbf{0} & {\mathbf{W}^{\prime\prime}} \\ \end{array} } \right]} \\ \end{array}$$

and

13 $$\begin{array}{*{20}c} {J = \left[ {\begin{array}{*{20}c} {{\mathbf{J}^{\prime}}} & \mathbf{0} \\ \mathbf{0} & {\mathbf{J}^{\prime\prime}} \\ \end{array} } \right]} \\ \end{array}$$

The matrix expression in Eq. 11 evaluates to (Appendix 2)

14 $$\begin{array}{*{20}c} {{{\varvec{\Sigma}}}_{{\left[ {\begin{array}{*{20}c} {{\mathbf{p^{\prime}}}} \\ {{\mathbf{p^{\prime\prime}}}} \\ \end{array} } \right]}} = r^{2} \left[ {\begin{array}{*{20}c} {\left( {{\mathbf{J}}^{{\prime{\text{T}}}} {\mathbf{W^{\prime}J^{\prime}}}} \right)^{ - 1} } & {\left( {{\mathbf{J}}^{{\prime{\text{T}}}} {\mathbf{W^{\prime}J^{\prime}}}} \right)^{ - 1} } \\ {\left( {{\mathbf{J}}^{{\prime{\text{T}}}} {\mathbf{W^{\prime}J^{\prime}}}} \right)^{ - 1} } & {\left( {{\mathbf{J}}^{{\prime\prime{\text{T}}}} {\mathbf{W}^{\prime\prime}}{\mathbf{J}^{\prime\prime}}}\right)^{ - 1} } \\ \end{array} } \right]} \\ \end{array}$$

Denote the Jacobian for the transformation to time integrated activities with ${{\varvec{\Upsilon}}}_{1}$ and ${{\varvec{\Upsilon}}}_{2}$ for ${\widetilde{A}}_{1}$ and ${\widetilde{A}}_{2}$, respectively. Under the assumption of unbiased estimates for the TAC parameters, ${{\varvec{\Upsilon}}}_{1}={{\varvec{\Upsilon}}}_{2}={\varvec{\Upsilon}}$. The covariance matrix for ${\widetilde{A}}_{1}$ and ${\widetilde{A}}_{2}$ becomes

15 $$\begin{array}{*{20}c} {{{\varvec{\Sigma}}}_{{\left[ {\begin{array}{*{20}c} {\tilde{A}_{1} } \\ {\tilde{A}_{2} } \\ \end{array} } \right]}} = \mathbf{U}{{\varvec{\Sigma}}}_{{\left[ {\begin{array}{*{20}c} {{\mathbf{p^{\prime}}}} \\ {{\mathbf{p^{\prime\prime}}}} \\ \end{array} } \right]}} {\mathbf{U}}^{{\text{T}}} } \\ \end{array}$$

where

16 $$\begin{array}{*{20}c} {\mathbf{U} = \left[ {\begin{array}{*{20}c} {{\varvec{\Upsilon}}} & \mathbf{0} \\ \mathbf{0} & {{\varvec{\Upsilon}}} \\ \end{array} } \right]} \\ \end{array}$$

Thus,

17 $$\begin{array}{*{20}c} {{{\varvec{\Sigma}}}_{{\left[ {\begin{array}{*{20}c} {\tilde{A}_{1} } \\ {\tilde{A}_{2} } \\ \end{array} } \right]}} = r^{2} \left[ {\begin{array}{*{20}c} {{{\varvec{\Upsilon}}}\left( {{\mathbf{J}}^{{{\prime}{\text{T}}}} {\mathbf{W^{\prime}J^{\prime}}}} \right)^{ - 1} {{\varvec{\Upsilon}}}^{{\text{T}}} } & {{{\varvec{\Upsilon}}}\left( {{\mathbf{J}}^{{{\prime}{\text{T}}}} {\mathbf{W^{\prime}J^{\prime}}}} \right)^{ - 1} {{\varvec{\Upsilon}}}^{{\text{T}}} } \\ {{{\varvec{\Upsilon}}}\left( {{\mathbf{J}}^{{{\prime}{\text{T}}}} {\mathbf{W^{\prime}J^{\prime}}}} \right)^{ - 1} {{\varvec{\Upsilon}}}^{{\text{T}}} } & {{{\varvec{\Upsilon}}}\left( {{\mathbf{J}}^{{{\prime\prime}{\text{T}}}} {\mathbf{W^{\prime}}}{^{\prime}}{\mathbf{J^{\prime}}}{^{\prime}}} \right)^{ - 1} {{\varvec{\Upsilon}}}^{{\text{T}}} } \\ \end{array} } \right]} \\ \end{array}$$

and the variance in Eq. 3 evaluates to

18 $$\begin{array}{*{20}c} {V\left[ \varepsilon \right] = E\left[ {\frac{{{{\varvec{\Upsilon}}}\left( {{\mathbf{J}}^{{{\prime}{\text{T}}}} {\mathbf{W^{\prime}J^{\prime}}}} \right)^{ - 1} {{\varvec{\Upsilon}}}^{{\text{T}}} }}{{\tilde{A}^{2} \left( {\mathbf{p}} \right)}}} \right]} \\ \end{array}$$

and the variance in Eq. 5 evaluates to

19 $$\begin{array}{*{20}c} {V\left[ \delta \right] = r^{2} E\left[ {\frac{{{{\varvec{\Upsilon}}}\left( {{\mathbf{J}}^{{{\prime\prime}{\text{T}}}} {\mathbf{W^{\prime}}}\user2{^{\prime}}{\mathbf{J^{\prime}}}\user2{^{\prime}}} \right)^{ - 1} {{\varvec{\Upsilon}}}^{{\text{T}}} - {{\varvec{\Upsilon}}}\left( {{\mathbf{J}}^{{{\prime}{\text{T}}}} {\mathbf{W^{\prime}J^{\prime}}}} \right)^{ - 1} {{\varvec{\Upsilon}}}^{{\text{T}}} }}{{ \tilde{A}^{2} \left( {\mathbf{p}} \right)}}} \right]} \\ \end{array}$$

### The mono-exponential case

Assume that

20 $$\begin{array}{*{20}c} {A\left( t \right) = A_{0} \exp \left[ { - \left( {\lambda_{{{\text{phys}}}} + \lambda_{{{\text{biol}}}} } \right)t} \right]} \\ \end{array}$$

where ${\lambda }_{\text{phys}}$ and ${\lambda }_{\text{biol}}$ are the physical and biological decay constants for the organ or tissue under consideration. Since ${\lambda }_{\text{phys}}$ does not depend on patient subject, the relevant parametrization of the TAC is $\mathbf{p}={\left[{A}_{0}\: {\lambda }_{\text{biol}}\right]}^{\text{T}}$. The Jacobian for the fittings of the TAC becomes

21 $$\begin{array}{*{20}c} {{\mathbf{J^{\prime}}} = \left[ {\begin{array}{*{20}c} {\frac{{\partial A\left( {t_{1} } \right)}}{{\partial A_{0} }}} & {\frac{{\partial A\left( {t_{1} } \right)}}{{\partial \lambda_{{{\text{biol}}}} }}} \\ {\frac{{\partial A\left( {t_{2} } \right)}}{{\partial A_{0} }}} & {\frac{{\partial A\left( {t_{2} } \right)}}{{\partial \lambda_{{{\text{biol}}}} }}} \\ {\begin{array}{*{20}c} \vdots \\ {\frac{{\partial A\left( {t_{n} } \right)}}{{\partial A_{0} }}} \\ \end{array} } & {\begin{array}{*{20}c} \vdots \\ {\frac{{\partial A\left( {t_{n} } \right)}}{{\partial \lambda_{{{\text{biol}}}} }}} \\ \end{array} } \\ \end{array} } \right] = \left[ {\begin{array}{*{20}c} {\frac{{A\left( {t_{1} } \right)}}{{A_{0} }}} & { - t_{1} A\left( {t_{1} } \right)} \\ {\frac{{A\left( {t_{2} } \right)}}{{A_{0} }}} & { - t_{2} A\left( {t_{2} } \right)} \\ \vdots & \vdots \\ {\frac{{A\left( {t_{n} } \right)}}{{A_{0} }}} & { - t_{n} A\left( {t_{n} } \right)} \\ \end{array} } \right]} \\ \end{array}$$

and

22 $$\begin{array}{*{20}c} {\mathbf{J}^{\prime\prime} = \left[ {\begin{array}{*{20}c} {\frac{{\partial A\left( {t_{1} } \right)}}{{\partial A_{0} }}} & {\frac{{\partial A\left( {t_{1} } \right)}}{{\partial \lambda_{{{\text{biol}}}} }}} \\ {\frac{{\partial A\left( {t_{2} } \right)}}{{\partial A_{0} }}} & {\frac{{\partial A\left( {t_{2} } \right)}}{{\partial \lambda_{{{\text{biol}}}} }}} \\ {\begin{array}{*{20}c} \vdots \\ {\frac{{\partial A\left( {t_{n - k} } \right)}}{{\partial A_{0} }}} \\ \end{array} } & {\begin{array}{*{20}c} \vdots \\ {\frac{{\partial A\left( {t_{n - k} } \right)}}{{\partial \lambda_{{{\text{biol}}}} }}} \\ \end{array} } \\ \end{array} } \right] = \left[ {\begin{array}{*{20}c} {\frac{{A\left( {t_{1} } \right)}}{{A_{0} }}} & { - t_{1} A\left( {t_{1} } \right)} \\ {\frac{{A\left( {t_{2} } \right)}}{{A_{0} }}} & { - t_{2} A\left( {t_{2} } \right)} \\ \vdots & \vdots \\ {\frac{{A\left( {t_{n - k} } \right)}}{{A_{0} }}} & { - t_{n - k} A\left( {t_{n - k} } \right)} \\ \end{array} } \right]} \\ \end{array}$$

The matrix expression ${\mathbf{J}}^{\mathbf{{\prime}}\text{T}}{\mathbf{W}}^{\mathbf{{\prime}}}{\mathbf{J}}^{\mathbf{{\prime}}}$ evaluates to

23 $$\begin{array}{*{20}c} {{\mathbf{J}}^{{{\prime}{\text{T}}}} {\mathbf{W^{\prime}J^{\prime}}} = n\left[ {\begin{array}{*{20}c} {A_{0}^{ - 2} } & { - A_{0}^{ - 1} \overline{{t^{\prime}}} } \\ { - A_{0}^{ - 1} \overline{{t^{\prime}}} } & {s^{{\prime}{2}} + \overline{{t^{\prime}}}^{2} } \\ \end{array} } \right]} \\ \end{array}$$

where

24 $$\begin{array}{*{20}c} {\overline{{t^{\prime}}} = \frac{1}{n}\mathop \sum \limits_{i = 1}^{n} t_{i} } \\ \end{array}$$

and

25 $$\begin{array}{*{20}c} {s^{{\prime}{2}} = \frac{1}{n}\mathop \sum \limits_{i = 1}^{n} \left( {t_{i} - \overline{t^{\prime}} } \right)^{2} } \\ \end{array}$$

The inverse of Eq. 23 is

26 $$\begin{array}{*{20}c} {\left( {{\mathbf{J}}^{{{\prime}{\text{T}}}} {\mathbf{W^{\prime}J^{\prime}}}} \right)^{ - 1} = \frac{{A_{0}^{2} }}{{ns^{{\prime}{2}} }}\left[ {\begin{array}{*{20}c} {s^{{\prime}{2}} + \overline{{t^{\prime}}}^{2} } & {A_{0}^{ - 1} \overline{{t^{\prime}}} } \\ {A_{0}^{ - 1} \overline{{t^{\prime}}} } & {A_{0}^{ - 2} } \\ \end{array} } \right]} \\ \end{array}$$

and, by analogy,

27 $$\begin{array}{*{20}c} {\left( {{\mathbf{J}}^{{{\prime\prime}{\text{T}}}} {\mathbf{W^{\prime}}}{^{\prime}}{\mathbf{J^{\prime}}}{^{\prime}}} \right)^{ - 1} = \frac{{A_{0}^{2} }}{{\left( {n - k} \right)s^{{\prime\prime}{2}} }}\left[ {\begin{array}{*{20}c} {s^{{\prime}{\prime}{2}} + \overline{{t^{\prime\prime}}}^{2} } & {A_{0}^{ - 1} \overline{{t^{\prime\prime}}} } \\ {A_{0}^{ - 1} \overline{{t^{\prime\prime}}} } & {A_{0}^{ - 2} } \\ \end{array} } \right]} \\ \end{array}$$

where

28 $$\begin{array}{*{20}c} {\overline{{t^{\prime\prime}}} = \frac{1}{n - k}\mathop \sum \limits_{i = 1}^{n - k} t_{i} } \\ \end{array}$$

and

29 $$\begin{array}{*{20}c} {s^{{\prime\prime}{2}} = \frac{1}{n - k}\mathop \sum \limits_{i = 1}^{n - k} \left( {t_{i} - \overline{{t^{\prime\prime}}} } \right)^{2} } \\ \end{array}$$

Hence, the covariance matrix for the TAC parameters becomes

30 $$\begin{array}{c}{{\varvec{\Sigma}}}_{\left[\begin{array}{c}{A}_{0}^{\prime}\\ {\lambda }_{\text{biol}}^{\prime}\\ \begin{array}{c}{A}_{0}^{{\prime}{\prime}}\\ {\lambda }_{\text{biol}}^{{\prime}{\prime}}\end{array}\end{array}\right]}={r}^{2}\left[\begin{array}{cccc}\frac{{A}_{0}^{2}}{n{s}^{{\prime}2}}\left({{s}^{\prime}}^{2}+{\overline{{t }^{\prime}}}^{2}\right)& \frac{{A}_{0}}{n{s}^{{\prime}2}}\overline{{t }^{\prime}}& \frac{{A}_{0}^{2}}{n{s}^{{\prime}2}}\left({{s}^{\prime}}^{2}+{\overline{{t }^{\prime}}}^{2}\right)& \frac{{A}_{0}}{n{s}^{{\prime}2}}\overline{{t }^{\prime}}\\ \frac{{A}_{0}}{n{s}^{{\prime}2}}\overline{{t }^{\prime}}& \frac{1}{n{s}^{{\prime}2}}& \frac{{A}_{0}}{n{s}^{{\prime}2}}\overline{{t }^{\prime}}& \frac{1}{n{s}^{{\prime}2}}\\ \frac{{A}_{0}^{2}}{n{s}^{{\prime}2}}\left({{s}^{\prime}}^{2}+{\overline{{t }^{\prime}}}^{2}\right)& \frac{{A}_{0}}{n{s}^{{\prime}2}}\overline{{t }^{\prime}}& \frac{{A}_{0}^{2}}{\left(n-k\right){s}^{{\prime}{\prime}2}}\left({{s}^{{\prime}{\prime}}}^{2}+{\overline{{t }^{\prime}}}^{2}\right)& \frac{{A}_{0}}{\left(n-k\right){s}^{{\prime}{\prime}2}}\overline{{t }^{{\prime}{\prime}}}\\ \frac{{A}_{0}}{n{s}^{{\prime}2}}\overline{{t }^{\prime}}& \frac{1}{n{s}^{{\prime}2}}& \frac{{A}_{0}}{\left(n-k\right){s}^{{\prime}{\prime}2}}\overline{{t }^{{\prime}{\prime}}}& \frac{1}{\left(n-k\right){s}^{{\prime}{\prime}2}}\end{array}\right].\end{array}$$

A few aspects can be noted from Eq. 30: ***a***) The CV of ${A}_{0}$ is $r\sqrt{\left(1+{\overline{{t }^{\prime}}}^{2}/{{s}^{\prime}}^{2}\right)/n}$. The CV is reduced by an increased number of data-points with a large dispersion in time (high${{s}{\prime}}^{2}$) and centred around $t=0$ (low$\overline{{t }{\prime}}$). ***b***) The standard deviation of the biological decay constant is $r\sqrt{1/\left(n{s}^{{\prime}2}\right)}$, which is decreased by a large number of measurement time-points and a large dispersion of time-points (large${s}^{{\prime}2}$). ***c***) ${A}_{0}$ and ${\lambda }_{\text{biol}}$ are positively correlated with correlation coefficient $\overline{{t }^{\prime}}/\sqrt{{{s}^{\prime}}^{2}+{\overline{{t }^{\prime}}}^{2}}$. ***d***) The two estimates of ${A}_{0}$ and biological decay constant are positively correlated, and the correlation coefficient is the ratio of the standard deviation for the estimate from the full dataset and the standard deviation for the estimate from the reduced dataset.

The Jacobian for the transformation to TIA for a mono-exponential TAC is

31 $$\begin{array}{*{20}c} {\mathbf{\Upsilon} = \tilde{A}\left[ {\begin{array}{*{20}c} {A_{0}^{ - 1} } & { - \tau } \\ \end{array} } \right]} \\ \end{array}$$

where the time constant $\tau ={\left({\lambda }_{\text{biol}}+{\lambda }_{\text{phys}}\right)}^{-1}$. Hence, the expression ${\varvec{\Upsilon}}{\left({\mathbf{J}}^{\mathbf{{\prime}}\text{T}}{\mathbf{W}}^{\mathbf{{\prime}}}{\mathbf{J}}^{\mathbf{{\prime}}}\right)}^{-1}{{\varvec{\Upsilon}}}^{\text{T}}$ evaluates to

32 $$\begin{array}{*{20}c} {\mathbf{\Upsilon} \left( {{\mathbf{J}}^{{{\prime}{\text{T}}}} {\mathbf{W^{\prime}J^{\prime}}}} \right)^{ - 1} {{\varvec{\Upsilon}}}^{{\text{T}}} = \frac{{\tilde{A}^{2} }}{n}\left[ {1 + \frac{{\left( {\overline{t^{\prime}} - \tau } \right)^{2} }}{{s^{^{\prime}2} }}} \right]} \\ \end{array}$$

and the variance in Eq. 18 becomes

33 $$\begin{array}{*{20}c} {V\left[ \varepsilon \right] = r^{2} \frac{1}{n}\left( {1 + \frac{{E\left[ {\left( {\overline{{t^{\prime}}} - \tau } \right)^{2} } \right]}}{{s^{{\prime}2} }}} \right) = r^{2} \frac{1}{n}\left( {1 + \frac{{\left( {\overline{{t^{\prime}}} - E\left[ \tau \right]} \right)^{2} }}{{s^{{\prime}2} }} + \frac{V\left[ \tau \right]}{{s^{{\prime}2} }}} \right)} \\ \end{array}$$

The covariance matrix in Eq. 17 evaluates to

34 $$\begin{array}{*{20}c} {\Sigma_{{\left[ {\begin{array}{*{20}c} {\tilde{A}_{1} } \\ {\tilde{A}_{2} } \\ \end{array} } \right]}} = r^{2} \tilde{A}^{2} \left[ {\begin{array}{*{20}c} {\frac{1}{n}\left[ {1 + \frac{{\left( {\overline{{t^{\prime}}} - \tau } \right)^{2} }}{{s^{{\prime}2} }}} \right]} & {\frac{1}{n}\left[ {1 + \frac{{\left( {\overline{{t^{\prime}}} - \tau } \right)^{2} }}{{s^{{\prime}2} }}} \right]} \\ {\frac{1}{n}\left[ {1 + \frac{{\left( {\overline{{t^{\prime}}} - \tau } \right)^{2} }}{{s^{{\prime}2} }}} \right]} & {\frac{1}{n - k}\left[ {1 + \frac{{\left( {\overline{{t^{\prime\prime}}} - \tau } \right)^{2} }}{{s^{{\prime{\prime 2} }} }}} \right]} \\ \end{array} } \right]} \\ \end{array}$$

and the variance in Eq. 19 becomes

35 $$\begin{array}{*{20}c} {V\left[ \delta \right] = r^{2} \left\{ {\frac{1}{n - k}\left[ {1 + \frac{{\left( {\overline{{t^{\prime\prime}}} - E\left[ \tau \right]} \right)^{2} }}{{s^{{\prime{\prime 2} }} }}} \right] - \frac{1}{n}\left[ {1 + \frac{{\left( {\overline{{t^{\prime}}} - E\left[ \tau \right]} \right)^{2} }}{{s^{{\prime}2} }}} \right] + \frac{V\left[ \tau \right]}{{\left( {n - k} \right)s^{{\prime{\prime 2} }} }} - \frac{V\left[ \tau \right]}{{ns^{{\prime}2} }}} \right\}} \\ \end{array}$$

The following observations can be made from Eq. 33 and Eq. 35: ***a***) For a given set of TAC parameters, the standard deviation in estimated TIA relative error is lowered by having a large number of measurement time-points (large *n*) with a large dispersion in time (large ${s}^{{\prime}2}$) centred around the time constant $\tau ={\left({\lambda }_{\text{biol}}+{\lambda }_{\text{phys}}\right)}^{-1}.$ ***b***) For a population of TAC parameters, the dispersion of errors is minimized by the same parameters as for the case of a given set of parameters, but the measurement time-points should be centred around the expectation of $\tau$ instead. As the dispersion of time constants increases, the dispersion of measurement time-points becomes relatively more important compared with the centring. ***c***) Two estimates of the TIA with the full and a reduced dataset are positively correlated with correlation coefficient being the ratio of the standard deviation of the full dataset and the reduced dataset.

To conclude, the variance of relative errors in TIA (*i.e.*, a measure of the precision in TIA estimates) should, theoretically, follow the pattern in Eq. 33, and is minimized by centring the measurement time-points around the mean of the inverse of the effective decay constant and by having a large dispersion (*i.e.*, variance) of time-points. Similarly, the variance of the relative difference between an estimate using the full dataset and a reduced dataset is predicted by Eq. 35. This is the variation that should be observed when comparing measurement schedules empirically by gradually removing time-points from an initial dataset.

## Methods

### Numerical verification

The analytical expression derived above were compared with estimates obtained using numerical simulations for two scenarios where the TAC parameters were either fixed or assumed to vary within the population. The scenarios were meant for verification of the analytical expressions using fewer assumptions than in the derivations. A summary of parameters used for the verifications are presented in Table 2.

**Table 2:** Summary of parameters used for the numerical validation

Parameter	Scenario 1: Fixed TAC	Scenario 2: Population of TACs
${A}_{0}$	100 MBq	100 MBq (with 50% population CV)
${T}_{\text{eff}}$	50 h	50 h (mean)
${\lambda }_{\text{biol}}$	0.009518 h^−1^	0.009518 h^−1^ (with 40% population CV)
CV of estimated activities	10%	10%
Fixed time points	4 h and 96 h	24 h, 96 h., 168 h; 24 h, 96 h.; or 96 h, 168 h p.i
Additional time point	0 h to 170 h (steps of 10 h)	0 h to 170 h (steps of 10 h)
Number of repetitions	10 000	10 000

#### Fixed TAC parameters

The underlying TAC for a ^177^Lu-labelled radiopharmaceutical (physical half-life 159.5 h (25)) was defined to have an ${A}_{0}=100\, \text{MBq}$ and an effective half-life of 50 h, corresponding to a biological decay constant of 0.009518 h^−1^. A CV of 10% was assumed for all estimated activities at the different time-points. Activities were in this scenario sampled at three time-points assuming log-normal distributions of estimated activities. Two of the time-points at 24 h and 96 h were kept fixed, whilst the third one was varied from 0 to 170 h in steps of 10 h with 10 000 repetitions per set of time-points. Simulations were performed in the IDL programming language (version 9.0, NV5 Geospatial solutions Inc.) using its built-in pseudo-random-number generator with standard normal Gaussian random numbers transformed to log-normally distributed ones with specified mean and variance. Time-activity curves were fitted using weighted non-linear least squares with Levenberg–Marquardt’s method in the mpfit library (26). Each data point was weighted with the inverse square of the estimated activity and the fitting was restricted such that both TAC parameters (${A}_{0}$ and biological decay-constant) were non-negative.

The numerically derived CVs for ${A}_{0}$, ${\lambda }_{\text{biol}}$, and $\widetilde{A}$ and the correlation between ${A}_{0}$ and ${\lambda }_{\text{biol}}$ were compared with the analytical values from Eq. 30 and Eq. 34.

#### Population of TAC parameters

A mean TAC for ^177^Lu with ${A}_{0}=100\, \text{MBq}$ and an effective half-life of 50 h was assumed with population CVs of 50% and 40% for ${A}_{0}$ and the biological decay constant, respectively. Numerical sampling and curve fitting was performed for measurement time-points at either 24 h, 96 h., and 168 h p.i.; 24 h, and 96 h p.i.; or 96 h, and 168 h p.i., assuming a CV of 10% for individual activity estimates. Fits were performed for these sets of fixed time-points and for the case with one extra time-point, varied between 0 and 170 h p.i., with simultaneous sampling of ${A}_{0}$ and ${\lambda }_{\text{biol}}$ assuming independent log-normal distributions.

The relative error of the TIA was calculated for each noise realization according to Eq. 2. To avoid the need for estimating the mean TAC for each noise realization, a bias-free estimation was assumed for the denominator. Likewise, for every fit to the full set of fixed and extra data-points a fit was performed using only the fixed time-points at and the relative deviation between the resulting two estimates of TIA was calculated according to Eq. 4. The standard deviation of relative errors and relative deviations were compared with their analytical counterparts according to Eq. 33 and Eq. 35.

### Patient data

Time activity-concentration data were retrieved for left kidney, right kidney, liver, and spleen for patients treated with ^177^Lu-DOTA-TATE from the study by Stenvall et al. (27). In brief, these data comprised 18 patients from the Iluminet trial (28) with activity concentrations estimated using hybrid planar-SPECT imaging. Data for left kidney were available from 17 patients, for right kidney from 18 patients, for liver from 18 patients, and for spleen from 16 patients.

Mono-exponential functions were fitted to data from 1 d, 4 d, and 7 d post-injection (p.i.) using weighted non-linear least squares with the Levenberg–Marquardt method (26) with data-points weighted as the inverse square of estimates. Curves were fitted using all three time-points and all combinations using only two time-points. The time-integrated activity concentrations estimated for the fits using two time-points were compared with the time-integrated activity concentrations estimated using all three time-points using Eq. 4 and the standard deviations calculated over patients.

As the CV for measurement data ($r$) was unknown, ratios of standard deviations were studied and compared with the ratios predicted by Eq. 35. The theoretical predictions were based on the mean time-points for which measurement data were reported and the mean and standard-deviations in time-constants $\tau$ from measurement data.

## Results

### Numerical verification

**Fig. 1:**
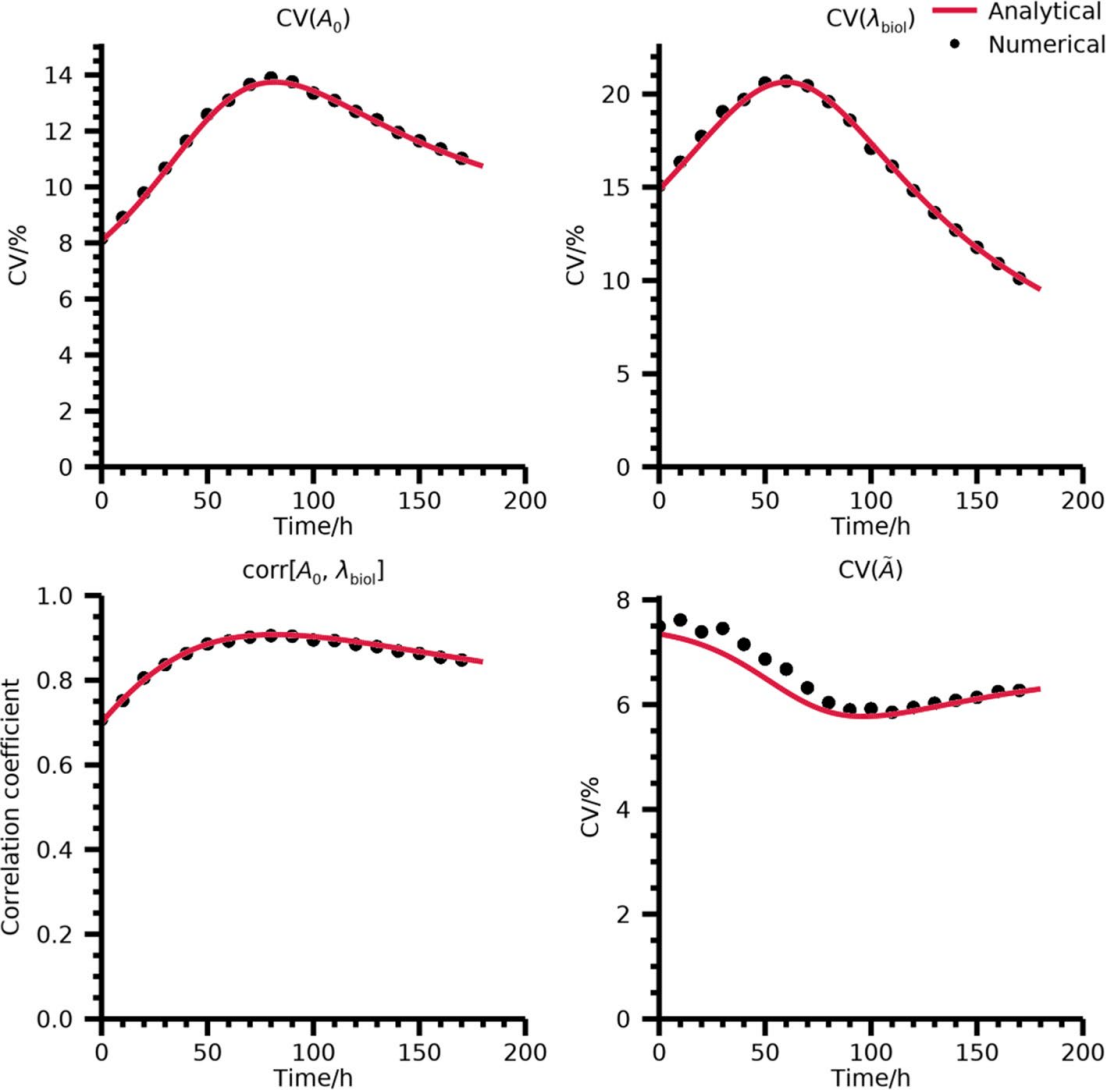
CVs for estimated ${A}_{0}$, $\lambda_{{{\text{biol}}}}$, the correlation between them, and CV for estimated TIA. Plots are for a mono-exponential function with effective half-life 50 h, physical half-life as for ^177^Lu, and CV of 10% in estimated activity

The agreement between CVs for ${A}_{0}$ and ${\lambda }_{\text{biol}}$ and the correlation between parameters calculated over noise and their analytical predictions are shown in Fig. 1. The agreement is shown as function of the third (mobile) imaging time-point. There is excellent agreement between analytical predictions and numerical simulations for the CVs of ${A}_{0}$ and ${\lambda }_{\text{biol}}$ and the correlation between them with deviations of 0.2 percentage points, 0.5 percentage points, and -0.008, respectively. For TIA, a maximum deviation of 0.5 percentage points is found. However, all trends for the numerically derived data are replicated by the analytical prediction. It is worth noting that the CV for TIA is lower than the CV for ${A}_{0}$ and ${\lambda }_{\text{biol}}$ individually. This is due to the positive correlation between these parameters resulting from the fitting procedure, and thus a partial cancelation of errors when the ratio between these parameters is formed. This also leads to the phenomenon that the combination of time-points that minimizes the CV for ${A}_{0}$ and ${\lambda }_{\text{biol}}$ individually does not minimize the CV in TIA.

**Fig. 2:**
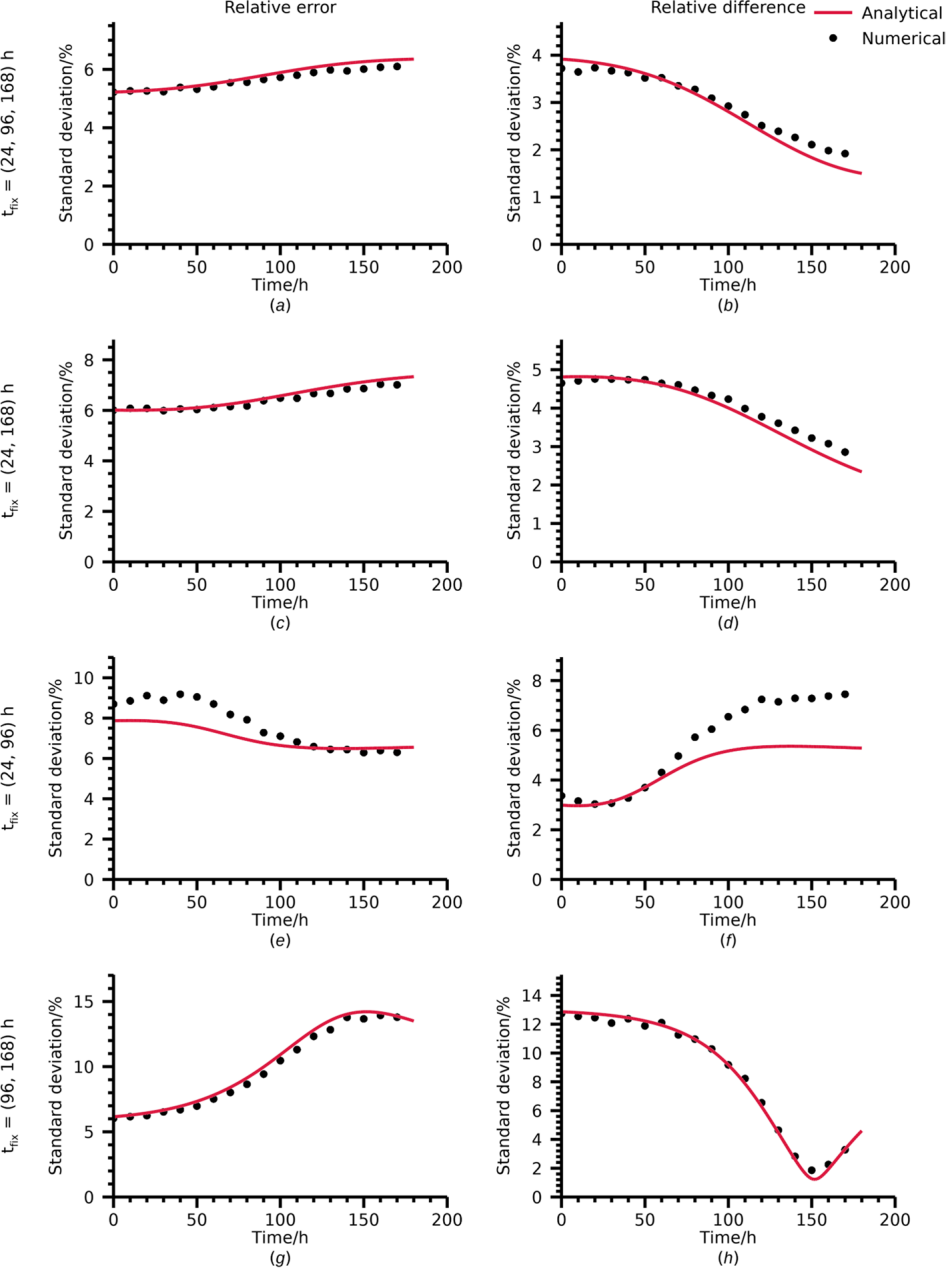
Standard deviations in relative errors and deviations as function of position of one added time-point. Four different cases are presented with underlying fixed time-points at 24 h, 96 h, and 168 h; 24 h and 168 h; 24 h and 96 h; and 96 and 168 h

The agreement between standard deviations for numerically derived errors and deviations and their analytical counterparts for the case with variable TAC parameters are shown in Fig. 2. For all combinations of fixed time-points, the trends demonstrated by the numerical experiments are replicated by the analytical predictions. In terms of numerical agreement, the errors differ by maximally -0.3 percentage points, -0.3 percentage points, 1.5 percentage points, and -0.7 percentage points for fixed time-points of 24 h, 96 h, and 168 h p.i.; 24 h, and 168 h p.i.; 24 h, and 96 h p.i.; and 96 h and 168 h p.i., respectively. Corresponding maximum differences for deviations between fits are 0.3 percentage points, 0.4 percentage points, 2.1 percentage points, and 0.6 percentage points.

### Patient data

Ratios between the standard deviations for the relative deviations for time-integrated activity-concentrations when using the first and second (${\sigma }_{12}$), and second and third time-points (${\sigma }_{23}$) for curve fitting compared with fitting using all three time-points relative to the standard deviation for first and third time-point (${\sigma }_{13}$) are presented in Table 3. The time constants derived from the reference fits (all three time-points) and used for the theoretical predictions are 81 h, 78 h, 111 h, and 110 h with CVs of 13%, 20%, 21%, and 22% for left kidney, right kidney, liver, and spleen, respectively. The mean time-points for which data were acquired were approximately 21 h, 93 h, and 165 h p.i. for all organs.

**Table 3:** Ratios between relative deviation standard deviations for different measurement schedules compared between the theory developed here and from experimental data

	Left kidney	Right kidney	Liver	Spleen				
	${\sigma }_{12}/{\sigma }_{13}$	${\sigma }_{23}/{\sigma }_{13}$	${\sigma }_{12}/{\sigma }_{13}$	${\sigma }_{23}/{\sigma }_{13}$	${\sigma }_{12}/{\sigma }_{13}$	${\sigma }_{23}/{\sigma }_{13}$	${\sigma }_{12}/{\sigma }_{13}$	${\sigma }_{23}/{\sigma }_{13}$
Theo	1.53	2.58	1.52	2.72	2.90	1.60	2.86	1.66
Exp	0.86	2.17	0.91	2.17	1.94	0.91	3.01	1.46

Since there are three time-points that could be removed, the four organs resulted in 12 cases in total. According to the analytical formulae developed in the present study, the standard deviation ratio ${\sigma }_{12}/{\sigma }_{13}$ should be lower than the ratio ${\sigma }_{23}/{\sigma }_{13}$ for kidneys, and larger for liver and spleen, which is also replicated by the empirical data. Furthermore, the theory predicts ratios larger than unity for all cases, which is replicated for the ratio ${\sigma }_{12}/{\sigma }_{13}$ for liver and spleen and the ratio ${\sigma }_{23}/{\sigma }_{13}$ for both kidneys and spleen. This means that theory correctly predicts the pairwise ordinal relations between the standard deviation when using time-point 1 and 2 versus time-point 2 and 3 for all cases. Similarly, the prediction is correct for time-point 1 and 2 versus time-point 1 and 3 for liver and spleen, and correct for time-point 2 and 3 versus time-point 1 and 3 for kidneys and spleen. In total, the theoretical order agreed with the experimental order in 9 out of 12 cases.

## Discussion

In this study analytical expressions were derived to analyse the influence of random variations in activity estimates and the propagation of those to a dispersion in TIA. The case of mono-exponential TACs was investigated to evaluate imaging schedules with reduced numbers of measurements. During recent years, the simplification of RNT measurement schemes has received substantial interest (3–6, 8–10, 15, 16), but generalisation and comparison of results is often difficult due to differences in imaging schedules or assessment criteria. We hope the consideration of the problem from a theoretical and analytical perspective will be useful in this regard and thereby facilitate the interpretation of patient and simulation studies.

One of the most important aspects of the current work is that the expressions derived for mono-exponential time-activity curves have straight-forward interpretations which may guide the development of optimal imaging schedules. The following properties should be noted: ***a***) The best-case propagation of errors for a single time-activity curve is be achieved if the average of all time-points is equal to the time-constant $\tau$. ***b***) Otherwise, the impact of the selection of time-points can be minimized by having a large dispersion of time-points. ***c***) With respect to a population of time activity curves, the variance can be decreased by ensuring the average of all time points used is equal to the mean time constant $\tau$ across the population. ***d***) However, the relative impact of the centring is decreased as the dispersion of time-constants in the population increases.

Since mono-exponential functions have an important role in many RNT dosimetry schemes, especially for imaging schedules with limited numbers of time-points, either alone, or in combination with other functions (22, 29–31), we believe these observations are useful for designing and optimizing dosimetry protocols. Optimal imaging schedules should have a large dispersion in time and be centred around the time-constant $\tau$. In practice, optimization in a clinical setting is also restricted by a number of other issues, such as camera availability, patient availability, and balancing between opposing objectives. For example, imaging during nights and weekends would be impossible for practical reasons, it is desirable to coordinate imaging with the patient’s other visits to the hospital, and often more than one structure may be of dosimetric interest for which optimal imaging schedules may differ. Furthermore, expanding these theoretical observations to specific case studies is complicated by the unavailability of effective half-lives for sufficiently large patient cohorts in the literature which would allow realistic determination of population effective half-lives and population CVs. Any proposed optimal imaging schedules in the present work would therefore be most likely be influenced by the uncertainties in the small datasets available. In addition, the variability in the effective half-lives will largely depend on the type of tissue of interest (*e.g.*, kidneys or lesions), the patient population in question and the radiopharmaceutical used and general recommendations are, therefore, difficult to obtain. A comprehensive overview of variabilities in effective half-lives in different forms of RNT have been presented by Hou et al. (32).

**Fig. 3:**
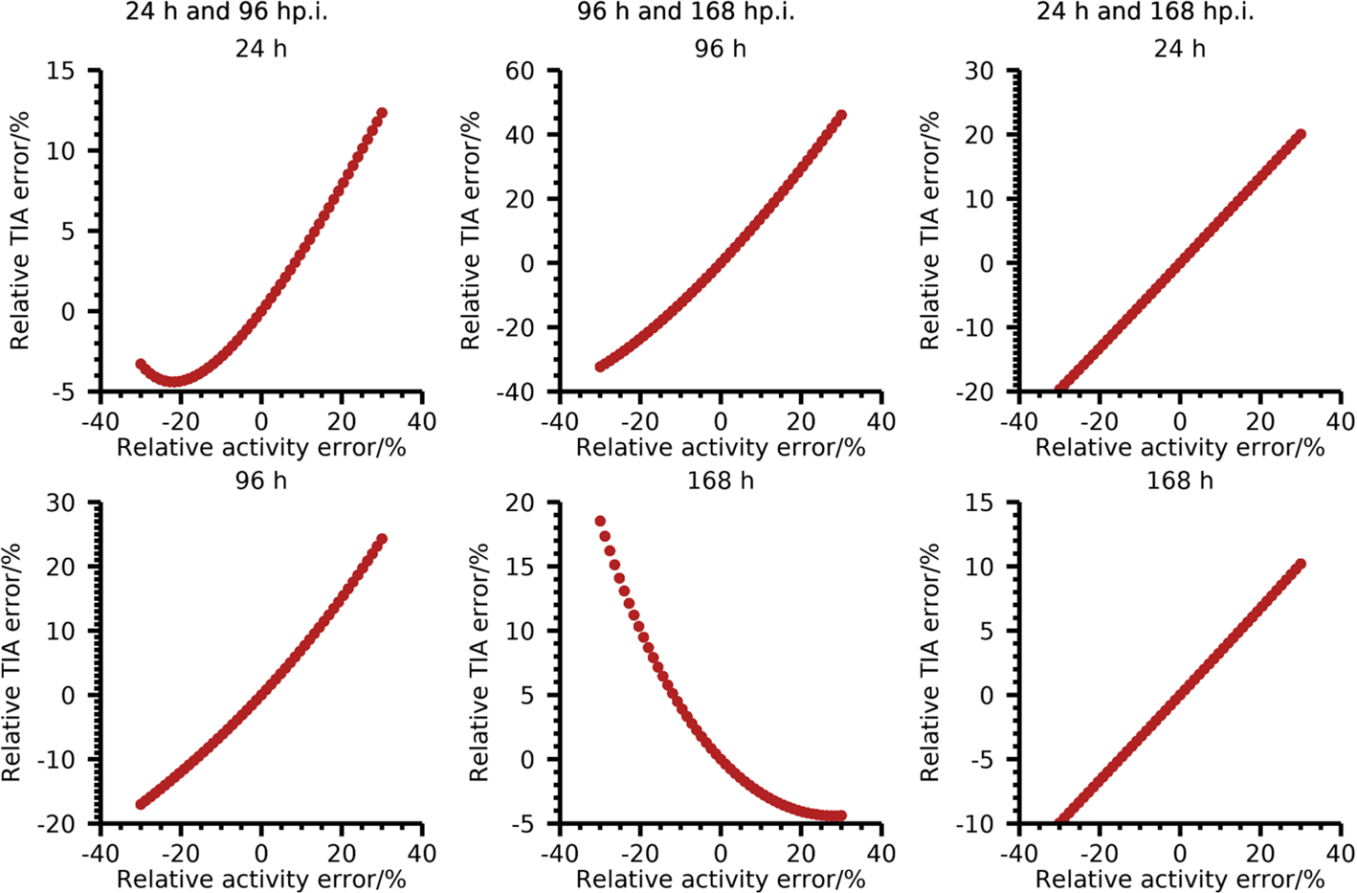
Relative TIA error as function of relative error in activity for one of the time-points. Plots are for three two-time-point fits

The analytical derivations performed are based on a number of assumptions and simplifications that may not necessarily be fulfilled in practice. Most importantly, the analytical expression for the TAC (*e.g.*, a mono-exponential function) is assumed to be known, the CV in estimated activity is assumed to be constant, and the weighting in the fit is assumed to be exactly inversely proportional to the variance of the activity estimate. To this also comes the linearization approximation inherent to the application of the LPU. Some of these simplifications are addressed by the numerical experiments performed. These experiments are similar to the strategy of Monte Carlo propagation of uncertainty (33) which relies on fewer assumptions than its analytical counterpart. In particular, the numerical verification allowed for relaxation of the linearization approximation and the assumption of ideal weighting, *i.e.*, that the weighting matrix was based on the time-activity curve itself, used in the analytical derivations. The CVs derived analytically and numerically were demonstrated to be in reasonable agreement with each other (Figs. 1 and 2), thereby giving credibility to the analytical expressions also for the case with less strict assumptions. The worst agreement for the specific population of effective half-lives studied was found for the relative error and the relative deviation for fixed time-points 24 h and 96 h p.i. (Fig. 2e and f). We believe this is mainly an effect of the linearization approximation for the fitting using the two fixed time-points. The linearization approximation is valid if a deviation in estimated activity leads to a proportional change in TIA. The error in estimated TIA as function of error in estimated activity for one time-point for fits based on 24 h and 96 h p.i.; 96 h and 168 h p.i.; and 24 h and 168 h p.i. are demonstrated in Fig. 3. As the LPU is based on a first-order Taylor expansion around the mean, these plots should ideally form straight lines for a good agreement between numerical simulations and analytical calculations. The pronounced non-linear behaviour for the 24 h time-point for the case with measurements at 24 h and 96 h p.i., in particular in comparison with the case of 24 h and 168 h p.i. Taken to its extreme, the Monte Carlo propagation of uncertainty can be expanded to a virtual clinical trial. Whilst such trials are more flexible in terms of what effects can be considered in the optimization of measurement schemes, they may become computationally intensive and are, in our experience, less able to provide an intuitive understanding of the problem at hand than an analytical formula. Hence, we argue that derivation using the LPU, whilst requiring more assumptions than numerical simulations, is a valuable complement to numerical approaches.

The derivation of the covariance between estimates using different number of time-points allowed us to relate the theoretical calculations of uncertainty to the common strategy of comparing differences between results from measurement schemes with one or several time-points removed (3, 5, 6, 8). Hence, we could compare the relative order of standard deviations predicted by theory with those determined empirically for a set of patients, and thereby, to some extent, assess the theory using real data without the assumption of the analytical derivations or numerical verifications. The relatively small sample size of 18 individuals makes the estimation of standard deviations from the sample in themselves relatively uncertain (34) and this initial demonstration can mainly serve as an example of how the formulae derived can be applied in an empirical context. Yet, the order of standard deviations predicted by theory is mostly the same as the order for the empirically determined ones. That is, the best scheme identified by the experimental data would also in most cases be the one predicted by theory. However, the comparison should preferably be reproduced for a much larger data material for a quantitative comparison to be more relevant. In addition to these purely statistical concerns, deviations between theory and experiments could also arise from deviations from the underlying assumptions of the theoretical predictions, for example, deviations from an underlying mono-exponential behaviour of the kinetics or deviation from the assumption of constant CV. Furthermore, the mean time-constants and their CVs used for the predictions were derived from the sample itself, which is not ideal. Hence, the theoretical predictions are unlikely to be a substitute for empirical comparisons, but should be able to function as a guide for designing experiments and as a lens through which results could be interpreted, thereby adding value to such studies.

There is still no generally established method for deriving the CV in estimated activity, or related quantities, from nuclear-medicine images, *i.e.*, the input $r$ for the theoretical framework derived in the current paper, and those papers who have studied the uncertainty of such data have mainly focused on systematic components (23, 35). However, as $r$ only acts as a multiplicative factor in Eq. 33 and Eq. 35, this value is not important for comparing measurement schemes in a relative sense. For those cases where also the absolute values are of interest, the CV in estimated activity can be estimated from the residual variance from the curve fitting.

The use of multi-exponential fitting functions even for a small number of time-points in RNT dosimetry has been explored in the literature (13). Nevertheless, in the current study the full evaluation and validation of formulae are limited to the simplest case of a mono-exponential TAC which in most cases remains the preferred choice of fitting function especially for imaging schedules with limited numbers of measurements. Nevertheless, the underlying principles for uncertainty propagation are applicable also to more complex TACs, and some preliminary results for generalization to the bi-exponential case can be found in the supplemental material to this paper. The full expressions are complicated, and any detailed intuitive understanding of the processes involved is limited, in contrary to the mono-exponential case. However, one can observe that the uncertainty is partitioned into distinctive terms associated with the fitting of each phase individually, corrections for the simultaneous fitting of the two phases, and the correlation between phases. Furthermore, the application of the general principles for uncertainty propagation are still applicable for bi- or multi-exponential functions, although then probably in a numerical setting rather than for derivation of explicit formulae.

The assumption of a fixed relative uncertainty for all estimated activities is reasonable if the major reasons for random errors are due to the processing to extract activity from reconstructed images (*e.g.*, image segmentation or co-registration) and that activity-dependent factors, *e.g.*, quantum noise, is of minor importance. For a given geometry, the effect of other random deviations, for example small differences in VOI definitions, should then scale with the activity in the structure considered. We believe this is a reasonable assumption for most cases of curve fitting in RNT dosimetry, where activities are typically high (15, 16, 23, 24, 36). However, for cases of very low administered activities (37), very short acquisition times (38), or measurements at very late time-points (39), this assumption might need to be reconsidered.

## Conclusions

The CV of time-integrated activities resulting from random errors in estimated activities can be theoretically studied using the law of propagation of uncertainty. For mono-exponential time-activity curves, this analysis predicts simple relationships for the propagation, which also seems to hold if verified numerically and when applied to patient data. Measurement time-points should, ideally, be centred such that their mean coincide with the mean of the inverse of the effective decay constant and should have an as high dispersion as possible. These findings may inform the development of imaging schedules for clinical trials and routine clinical dosimetry of new or established radiopharmaceuticals.

## Supplementary Information

Additional file 1.

## Data Availability

Data are available from the corresponding author on reasonable request.

## Appendix 1

The estimation of fitting parameters for the full and reduced data set can be set up as the (overdetermined) equation system

$$\left[ {\begin{array}{*{20}c} {A_{1} } \\ {A_{2} } \\ {\begin{array}{*{20}c} \vdots \\ {A_{n} } \\ {\begin{array}{*{20}c} {A_{1} } \\ {A_{2} } \\ {\begin{array}{*{20}c} \vdots \\ {A_{n - k} } \\ \end{array} } \\ \end{array} } \\ \end{array} } \\ \end{array} } \right] = \left[ {\begin{array}{*{20}c} {A\left( {t_{1} ;{\mathbf{p^{\prime}}}} \right)} \\ {A\left( {t_{2} ;{\mathbf{p^{\prime}}}} \right)} \\ {\begin{array}{*{20}c} \vdots \\ {A\left( {t_{n} ;{\mathbf{p^{\prime}}}} \right)} \\ {\begin{array}{*{20}c} {A\left( {t_{1} ;{\mathbf{p^{\prime\prime}}}} \right)} \\ {A\left( {t_{2} ;{\mathbf{p^{\prime\prime}}}} \right)} \\ {\begin{array}{*{20}c} \vdots \\ {A\left( {t_{n - k} ;{\mathbf{p^{\prime\prime}}}} \right)} \\ \end{array} } \\ \end{array} } \\ \end{array} } \\ \end{array} } \right] \approx \left[ {\begin{array}{*{20}c} {A\left( {t_{1} ;{\mathbf{p}}} \right)} \\ {A\left( {t_{2} ;{\mathbf{p}}} \right)} \\ {\begin{array}{*{20}c} \vdots \\ {A\left( {t_{n} ;{\mathbf{p}}} \right)} \\ {\begin{array}{*{20}c} {A\left( {t_{1} ;{\mathbf{p}}} \right)} \\ {A\left( {t_{2} ;{\mathbf{p}}} \right)} \\ {\begin{array}{*{20}c} \vdots \\ {A\left( {t_{n - k} ;{\mathbf{p}}} \right)} \\ \end{array} } \\ \end{array} } \\ \end{array} } \\ \end{array} } \right] + \left[ {\begin{array}{*{20}c} {\mathop \sum \limits_{j = 1}^{m} \frac{{\partial A\left( {t_{1} } \right)}}{{\partial p_{j} }}\left( {p_{j} ^{\prime} - p_{j} } \right)} \\ {\mathop \sum \limits_{j = 1}^{m} \frac{{\partial A\left( {t_{2} } \right)}}{{\partial p_{j} }}\left( {p_{j} ^{\prime} - p_{j} } \right)} \\ {\begin{array}{*{20}c} \vdots \\ {\mathop \sum \limits_{j = 1}^{m} \frac{{\partial A\left( {t_{n} } \right)}}{{\partial p_{j} }}\left( {p_{j} ^{\prime} - p_{j} } \right)} \\ {\begin{array}{*{20}c} {\mathop \sum \limits_{j = 1}^{m} \frac{{\partial A\left( {t_{1} } \right)}}{{\partial p_{j} }}\left( {p_{j} ^{\prime\prime} - p_{j} } \right)} \\ {\mathop \sum \limits_{j = 1}^{m} \frac{{\partial A\left( {t_{2} } \right)}}{{\partial p_{j} }}\left( {p_{j} ^{\prime\prime} - p_{j} } \right)} \\ {\begin{array}{*{20}c} \vdots \\ {\mathop \sum \limits_{j = 1}^{m} \frac{{\partial A\left( {t_{n - k} } \right)}}{{\partial p_{j} }}\left( {p_{j} ^{\prime\prime} - p_{j} } \right)} \\ \end{array} } \\ \end{array} } \\ \end{array} } \\ \end{array} } \right]$$

A1.1 $$\begin{array}{c}\iff \left[\begin{array}{c}{A}_{1}-A\left({t}_{1};\mathbf{p}\right)\\ {A}_{2}-A\left({t}_{2};\mathbf{p}\right)\\ \begin{array}{c}\vdots \\ {A}_{n}-A\left({t}_{n};\mathbf{p}\right)\\ \begin{array}{c}{A}_{1}-A\left({t}_{1};\mathbf{p}\right)\\ {A}_{2}-A\left({t}_{2};\mathbf{p}\right)\\ \begin{array}{c}\vdots \\ {A}_{n-k}-A\left({t}_{n-k};\mathbf{p}\right)\end{array}\end{array}\end{array}\end{array}\right]=\\ \left[\begin{array}{cccccccc}\frac{\partial A\left({t}_{1}\right)}{\partial {p}_{1}}& \frac{\partial A\left({t}_{1}\right)}{\partial {p}_{2}}& \cdots & \frac{\partial A\left({t}_{1}\right)}{\partial {p}_{m}}& 0& 0& \cdots & 0\\ \frac{\partial A\left({t}_{2}\right)}{\partial {p}_{1}}& \frac{\partial A\left({t}_{2}\right)}{\partial {p}_{2}}& \cdots & \frac{\partial A\left({t}_{2}\right)}{\partial {p}_{m}}& 0& 0& \cdots & 0\\ \vdots & \vdots & \ddots & \vdots & \vdots & \vdots & \ddots & \vdots \\ \frac{\partial A\left({t}_{n}\right)}{\partial {p}_{1}}& \frac{\partial A\left({t}_{n}\right)}{\partial {p}_{2}}& \cdots & \frac{\partial A\left({t}_{n}\right)}{\partial {p}_{m}}& 0& 0& \cdots & 0\\ 0& 0& \cdots & 0& \frac{\partial A\left({t}_{1}\right)}{\partial {p}_{1}}& \frac{\partial A\left({t}_{1}\right)}{\partial {p}_{2}}& \cdots & \frac{\partial A\left({t}_{1}\right)}{\partial {p}_{m}}\\ 0& 0& \cdots & 0& \frac{\partial A\left({t}_{2}\right)}{\partial {p}_{1}}& \frac{\partial A\left({t}_{2}\right)}{\partial {p}_{2}}& \cdots & \frac{\partial A\left({t}_{2}\right)}{\partial {p}_{m}}\\ \vdots & \vdots & \ddots & \vdots & \vdots & \vdots & \ddots & \vdots \\ 0& 0& \cdots & 0& \frac{\partial A\left({t}_{n-k}\right)}{\partial {p}_{1}}& \frac{\partial A\left({t}_{n-k}\right)}{\partial {p}_{2}}& \cdots & \frac{\partial A\left({t}_{n-k}\right)}{\partial {p}_{m}}\end{array}\right]\left[\begin{array}{c}{p}_{1}^{\prime}-{p}_{1}\\ {p}_{2}^{\prime}-{p}_{2}\\ \vdots \\ {p}_{m}^{\prime}-{p}_{m}\\ {p}_{1}^{\prime\prime} -{p}_{1}\\ {p}_{2}^{\prime\prime} -{p}_{2}\\ \vdots \\ {p}_{m}^{\prime\prime} -{p}_{m}\end{array}\right]\\ =\mathbf{J}\left[\begin{array}{c}{\mathbf{p}}^{\prime}-\mathbf{p}\\ {\mathbf{p}}^{{\prime}{\prime}}-\mathbf{p}\end{array}\right]\\ \iff \mathbf{A}-\mathbf{A}\left(\mathbf{t};\mathbf{p}\right)=\mathbf{J}\left[\begin{array}{c}{\mathbf{p}}^{\prime}-\mathbf{p}\\ {\mathbf{p}}^{{\prime}{\prime}}-\mathbf{p}\end{array}\right]\end{array}$$

Equation (A1.1) is a linear equation system for which the covariance matrix of the weighted least-squares solution is.

A1.2 $$\begin{array}{c}{{\varvec{\Sigma}}}_{\left[\begin{array}{c}{\mathbf{p}}^{\prime}-\mathbf{p}\\ {\mathbf{p}}^{{\prime}{\prime}}-\mathbf{p}\end{array}\right]}={\left({\mathbf{J}}^{\text{T}}\mathbf{W}\mathbf{J}\right)}^{-1}{\mathbf{J}}^{\text{T}}\mathbf{W}{{\varvec{\Sigma}}}_{\mathbf{A}-\mathbf{A}\left(\mathbf{t};\mathbf{p}\right)}\mathbf{WJ}{\left({\mathbf{J}}^{\text{T}}\mathbf{W}\mathbf{J}\right)}^{-1}.\end{array}$$

Since $\mathbf{p}$ and $\mathbf{A}\left(\mathbf{t};\mathbf{p}\right)$ are constants $${{\varvec{\Sigma}}}_{\left[\begin{array}{c}{\mathbf{p}}^{\prime}-\mathbf{p}\\ {\mathbf{p}}^{{\prime}{\prime}}-\mathbf{p}\end{array}\right]}={{\varvec{\Sigma}}}_{\left[\begin{array}{c}{\mathbf{p}}^{\prime}\\ {\mathbf{p}}^{{\prime}{\prime}}\end{array}\right]}$$ and ${{\varvec{\Sigma}}}_{\mathbf{A}-\mathbf{A}\left(\mathbf{t};\mathbf{p}\right)}={{\varvec{\Sigma}}}_{\mathbf{A}}$. Thus, (11) is obtained.

## Appendix 2

The matrix expression ${\mathbf{J}}^{\text{T}}\mathbf{W}{{\varvec{\Sigma}}}_{\mathbf{A}}\mathbf{W}\mathbf{J}$ evaluates to.

A2.1 $$\begin{aligned} {\mathbf{J}}^{{\text{T}}} {\mathbf{W\Sigma }}_{{\mathbf{A}}} {\mathbf{WJ}} = & \left[ {\begin{array}{*{20}c} {{\mathbf{J^{\prime}}}^{{\text{T}}} } & {\mathbf{0}} \\ {\mathbf{0}} & {{\mathbf{J}}^{{\prime\prime{\text{T}}}} } \\ \end{array} } \right]\left[ {\begin{array}{*{20}c} {{\mathbf{W^{\prime}}}} & {\mathbf{0}} \\ {\mathbf{0}} & {{\mathbf{W^{\prime\prime}}}} \\ \end{array} } \right]r^{2} \left[ {\begin{array}{*{20}c} {{\mathbf{W^{\prime}}}^{{ - 1}} } & {\left[ {\begin{array}{*{20}c} {{\mathbf{W^{\prime\prime}}}^{{ - 1}} } \\ {\mathbf{0}} \\ \end{array} } \right]} \\ {\left[ {\begin{array}{*{20}c} {{\mathbf{W^{\prime\prime}}}^{{ - 1}} } & {\mathbf{0}} \\ \end{array} } \right]} & {{\mathbf{W^{\prime\prime}}}^{{ - 1}} } \\ \end{array} } \right]\left[ {\begin{array}{*{20}c} {{\mathbf{W^{\prime}}}} & {\mathbf{0}} \\ {\mathbf{0}} & {{\mathbf{W^{\prime\prime}}}} \\ \end{array} } \right]\left[ {\begin{array}{*{20}c} {{\mathbf{J^{\prime}}}} & {\mathbf{0}} \\ {\mathbf{0}} & {{\mathbf{J^{\prime\prime}}}} \\ \end{array} } \right] \\ = & r^{2} \left[ {\begin{array}{*{20}c} {{\mathbf{J}}^{{\prime{\text{T}}}} {\mathbf{W^{\prime}J^{\prime}}}} & {{\mathbf{J}}^{{\prime\prime{\text{T}}}} {\mathbf{W}}^{\prime\prime}{\mathbf{J^{\prime\prime}}}} \\ {{\mathbf{J}}^{{\prime\prime{\text{T}}}} {\mathbf{W}}^{\prime\prime}{\mathbf{J^{\prime\prime}}}} & {{\mathbf{J}}^{{\prime\prime{\text{T}}}} {\mathbf{W}}^{\prime\prime}{\mathbf{J^{\prime\prime}}}} \\ \end{array} } \right], \\ \end{aligned}$$

Where the key is to identify that $${{\mathbf{J}}^{\prime}}^{\text{T}}\left[\begin{array}{c}{\mathbf{W}}^{\prime\prime}\\ \mathbf{0}\end{array}\right]={\mathbf{J}}^{\boldsymbol{{\prime}}\boldsymbol{{\prime}}\text{T}}{\mathbf{W}}^{\prime\prime}$$ and $$\left[\begin{array}{cc}{\mathbf{J}}^{\boldsymbol{{\prime}}\boldsymbol{{\prime}}\text{T}}{\mathbf{W}}^{{\prime}{\prime}}& \mathbf{0}\end{array}\right]{\mathbf{J}}^{\prime}={\mathbf{J}}^{\boldsymbol{{\prime}}\boldsymbol{{\prime}}\text{T}}{\mathbf{W}}^{{\prime}{\prime}}{\mathbf{J}}^{{\prime}{\prime}}$$.

The expression ${\left({\mathbf{J}}^{\text{T}}\mathbf{W}\mathbf{J}\right)}^{-1}$ evaluates to.

A2.2 $$\begin{aligned} \left( {{\mathbf{J}}^{{\text{T}}} {\mathbf{WJ}}} \right)^{{ - 1}} & = \left( {\left[ {\begin{array}{*{20}c} {{\mathbf{J^{\prime}}}^{{\text{T}}} } & \mathbf{0} \\ \mathbf{0} & {{\mathbf{J}}^{{\prime\prime{\text{T}}}} } \\ \end{array} } \right]\left[ {\begin{array}{*{20}c} {{\mathbf{W^{\prime}}}} & \mathbf{0} \\ 0 & {{\mathbf{W^{\prime\prime}}}} \\ \end{array} } \right]\left[ {\begin{array}{*{20}c} {{\mathbf{J^{\prime}}}} & \mathbf{0} \\ \mathbf{0} & {{\mathbf{J^{\prime\prime}}}} \\ \end{array} } \right]} \right)^{{ - 1}} \\ & = \left[ {\begin{array}{*{20}c} {{\mathbf{J^{\prime}}}^{{\text{T}}} {\mathbf{W^{\prime}J^{\prime}}}} & \mathbf{0} \\ \mathbf{0} & {{\mathbf{J}}^{{\prime\prime{\text{T}}}} {\mathbf{W^{\prime\prime}}}{\mathbf{J^{\prime\prime}}}} \\ \end{array} } \right]^{{ - 1}} \\ & = \left[ {\begin{array}{*{20}c} {\left( {{\mathbf{J^{\prime}}}^{{\text{T}}} {\mathbf{W^{\prime}J^{\prime}}}} \right)^{{ - 1}} } & \mathbf{0} \\ \mathbf{0} & {\left( {{\mathbf{J}}^{{\prime\prime{\text{T}}}} {\mathbf{W^{\prime\prime}}}{\mathbf{J^{\prime\prime}}}} \right)^{{ - 1}} } \\ \end{array} } \right] \\ \end{aligned}$$

Hence,

A2.3 $$\begin{aligned} {\mathbf{\Sigma }}_{{\left[ {\begin{array}{*{20}c} {{\mathbf{p^{\prime}}}} \\ {{\mathbf{p^{\prime\prime}}}} \\ \end{array} } \right]}} & = r^{2} \left[ {\begin{array}{*{20}c} {\left( {{\mathbf{J^{\prime}}}^{{\text{T}}} {\mathbf{W^{\prime}J^{\prime}}}} \right)^{{ - 1}} } & \mathbf{0} \\ \mathbf{0} & {\left( {{\mathbf{J}}^{{\prime\prime{\text{T}}}} {\mathbf{W^{\prime\prime}}}{\mathbf{J^{\prime\prime}}}} \right)^{{ - 1}} } \\ \end{array} } \right]\left[ {\begin{array}{*{20}c} {{\mathbf{J}}^{{\prime{\text{T}}}} {\mathbf{W^{\prime}J^{\prime}}}} & {{\mathbf{J}}^{{\prime\prime{\text{T}}}} {\mathbf{W^{\prime\prime}}} {\mathbf{J^{\prime\prime}}}} \\ {{\mathbf{J}}^{{\prime\prime{\text{T}}}} {\mathbf{W^{\prime\prime}}} {\mathbf{J^{\prime\prime}}}} & {{\mathbf{J}}^{{\prime\prime{\text{T}}}} {\mathbf{W^{\prime\prime}}} {\mathbf{J^{\prime\prime}}}} \\ \end{array} } \right]\left[ {\begin{array}{*{20}c} {\left( {{\mathbf{J^{\prime}}}^{{\text{T}}} {\mathbf{W^{\prime}J^{\prime}}}} \right)^{{ - 1}} } & 0 \\ 0 & {\left( {{\mathbf{J}}^{{\prime\prime{\text{T}}}} {\mathbf{W^{\prime\prime}}}{\mathbf{J^{\prime\prime}}}} \right)^{{ - 1}} } \\ \end{array} } \right] \\ & = r^{2} \left[ {\begin{array}{*{20}c} {\left( {{\mathbf{J^{\prime}}}^{{\text{T}}} {\mathbf{W^{\prime}J^{\prime}}}} \right)^{{ - 1}} } & {\left( {{\mathbf{J^{\prime}}}^{{\text{T}}} {\mathbf{W^{\prime}J^{\prime}}}} \right)^{{ - 1}} } \\ {\left( {{\mathbf{J^{\prime}}}^{{\text{T}}} {\mathbf{W^{\prime}J^{\prime}}}} \right)^{{ - 1}} } & {\left( {{\mathbf{J}}^{{\prime\prime{\text{T}}}} {\mathbf{W^{\prime\prime}}}{\mathbf{J^{\prime\prime}}}} \right)^{{ - 1}} } \\ \end{array} } \right] \\ \end{aligned}$$

## Abbreviations

CV: Coefficient of variation
LPU: Law of propagation of uncertainty
p.i.: Post injection
RNT: Radionuclide therapy
TAC: Time-activity curve
TIA: Time-integrated activity
