# Supplementary material for: On the reduction of imaging time-points for dosimetry in radionuclide therapy
Source: EJNMMI Phys. 2025 Feb 6;12:14. doi: 10.1186/s40658-025-00721-y (PMC11799503; doi:10.1186/s40658-025-00721-y)
Supplement: Supplementary file 1 — Additional file 1. [file 40658_2025_721_MOESM1_ESM.pdf]

Supplement to: On the reduction of imaging time-points for dosimetry in radionuclide therapy

Johan Gustafsson[1] and Jan Taprogge[2,3]

1. Medical Radiation Physics, Lund, Lund University, Lund, Sweden
2. Joint Department of Physics, Royal Marsden NHSFT, United Kingdom
3. The Institute of Cancer Research, London, United Kingdom

The bi-exponential case

Consider a bi-exponential TAC, *i.e.*,

$$A(t) = a_1 \exp\left(-\left[\lambda_1 + \lambda_{\text{phys}}\right]t\right) + a_2 \exp\left(-\left[\lambda_2 + \lambda_{\text{phys}}\right]t\right) \tag{S1}$$

corresponding to the parameter vector $\mathbf{p} = [a_1, \lambda_1, a_2, \lambda_2]^{\mathrm{T}}$.

The Jacobian for the fitting problem to the n data points is

$$\mathbf{J} = \begin{bmatrix} \exp\left(-\left[\lambda_1 + \lambda_{\text{phys}}\right]t_1\right) & -t_1 a_1 \exp\left(-\left[\lambda_1 + \lambda_{\text{phys}}\right]t_1\right) & \exp\left(-\left[\lambda_2 + \lambda_{\text{phys}}\right]t_1\right) & -t_1 a_2 \exp\left(-\left[\lambda_2 + \lambda_{\text{phys}}\right]t_1\right) \\ \exp\left(-\left[\lambda_1 + \lambda_{\text{phys}}\right]t_2\right) & -t_2 a_1 \exp\left(-\left[\lambda_1 + \lambda_{\text{phys}}\right]t_2\right) & \exp\left(-\left[\lambda_2 + \lambda_{\text{phys}}\right]t_2\right) & -t_2 a_2 \exp\left(-\left[\lambda_2 + \lambda_{\text{phys}}\right]t_2\right) \\ \vdots & \vdots & \vdots & \vdots \\ \exp\left(-\left[\lambda_1 + \lambda_{\text{phys}}\right]t_n\right) & -t_n a_1 \exp\left(-\left[\lambda_1 + \lambda_{\text{phys}}\right]t_n\right) & \exp\left(-\left[\lambda_2 + \lambda_{\text{phys}}\right]t_n\right) & -t_n a_2 \exp\left(-\left[\lambda_2 + \lambda_{\text{phys}}\right]t_n\right) \end{bmatrix} \tag{S2}$$

and the covariance matrix of the parameter vector becomes

$$\boldsymbol{\Sigma}_{\mathbf{p}} = r^2 \begin{bmatrix} Q_1 & -a_1 Q_1 \bar{t}_1 & Q_{12} & -a_2 Q_{12} \bar{t}_{12} \\ -a_1 Q_1 \bar{t}_1 & a_1^2 Q_1 (s_1^2 + \bar{t}_1^2) & -a_1 Q_{12} \bar{t}_{12} & a_1 a_2 Q_{12} (s_{12}^2 + \bar{t}_{12}^2) \\ Q_{12} & -a_1 Q_{12} \bar{t}_{12} & Q_2 & -a_2 Q_2 \bar{t}_2 \\ -a_2 Q_{12} \bar{t}_{12} & a_1 a_2 Q_{12} (s_{12}^2 + \bar{t}_{12}^2) & -a_2 Q_2 \bar{t}_2 & a_2^2 Q_2 (s_2^2 + \bar{t}_2^2) \end{bmatrix}^{-1} \tag{S3}$$

where $Q_1 = \sum_{i=1}^{n} q_1^2(t_i)$, $Q_2 = \sum_{i=1}^{n} q_2^2(t_i)$, and $Q_{12} = \sum_{i=1}^{n} q_1(t_i) q_2(t_i)$ with $q_1(t) = \exp\left(-\left[\lambda_1 + \lambda_{\text{phys}}\right]t\right)/A(t)$ and $q_2(t) = \exp\left(-\left[\lambda_2 + \lambda_{\text{phys}}\right]t\right)/A(t)$; $\bar{t}_1$, $\bar{t}_2$, and $\bar{t}_{12}$ are the weighted averages $\bar{t}_1 = \sum_{i=1}^{n} q_1^2(t_i) t_i / Q_1$, $\bar{t}_2 = \sum_{i=1}^{n} q_2^2(t_i) t_i / Q_2$ and $\bar{t}_{12} = \sum_{i=1}^{n} q_1(t_i) q_2(t_i) / Q_{12}$; and s_1^2, s_2^2, and s_{12}^2 are the weighted variances $s_1^2 = \sum_{i=1}^{n} q_1^2(t_i)(t_i - \bar{t}_1)^2 / Q_1$, $s_2^2 = \sum_{i=1}^{n} q_2^2(t_i)(t_i - \bar{t}_2)^2 / Q_2$, and $s_{12}^2 = \sum_{i=1}^{n} q_1(t_i) q_2(t_i)(t_i - \bar{t}_{12})^2 / Q_{12}$.

Denote

$$\boldsymbol{\Delta}_1 = \begin{bmatrix} Q_1 & -a_1 Q_1 \bar{t}_1 \\ -a_1 Q_1 \bar{t}_1 & a_1^2 Q_1 (s_1^2 + \bar{t}_1^2) \end{bmatrix}, \tag{S4}$$

$$\boldsymbol{\Delta}_2 = \begin{bmatrix} Q_2 & -a_2 Q_2 \bar{t}_2 \\ -a_2 Q_2 \bar{t}_2 & a_2^2 Q_2 (s_2^2 + \bar{t}_2^2) \end{bmatrix}, \tag{S5}$$

and

$$\mathbf{K} = \begin{bmatrix} Q_{12} & -a_2 Q_{12}\bar{t}_{12} \\ -a_1 Q_{12}\bar{t}_{12} & a_1 a_2 Q_{12}(s_{12}^2 + \bar{t}_{12}^2) \end{bmatrix} \quad \text{(S6)}$$

such that

$$\mathbf{\Sigma_P} = r^2 \begin{bmatrix} \mathbf{\Delta}_1 & \mathbf{K} \\ \mathbf{K}^{\mathrm{T}} & \mathbf{\Delta}_2 \end{bmatrix}^{-1} =$$
$$r^2 \begin{bmatrix} \mathbf{\Delta}_1^{-1} + \mathbf{\Delta}_1^{-1}\mathbf{K}(\mathbf{\Delta}_2 - \mathbf{K}^{\mathrm{T}}\mathbf{\Delta}_1^{-1}\mathbf{K})^{-1}\mathbf{K}^{\mathrm{T}}\mathbf{\Delta}_1^{-1} & -\mathbf{\Delta}_1^{-1}\mathbf{K}(\mathbf{\Delta}_2 - \mathbf{K}^{\mathrm{T}}\mathbf{\Delta}_1^{-1}\mathbf{K})^{-1} \\ -(\mathbf{\Delta}_2 - \mathbf{K}^{\mathrm{T}}\mathbf{\Delta}_1^{-1}\mathbf{K})^{-1}\mathbf{K}^{\mathrm{T}}\mathbf{\Delta}_1^{-1} & \mathbf{\Delta}_2^{-1} + \mathbf{\Delta}_2^{-1}\mathbf{K}^{\mathrm{T}}(\mathbf{\Delta}_1 - \mathbf{K}\mathbf{\Delta}_2^{-1}\mathbf{K}^{\mathrm{T}})^{-1}\mathbf{K}\mathbf{\Delta}_2^{-1} \end{bmatrix} =$$
$$r^2 \begin{bmatrix} \mathbf{\Delta}_1^{-1} & \mathbf{0} \\ \mathbf{0} & \mathbf{\Delta}_2^{-1} \end{bmatrix} + r^2 \begin{bmatrix} \mathbf{\Delta}_1^{-1}\mathbf{K}(\mathbf{\Delta}_2 - \mathbf{K}^{\mathrm{T}}\mathbf{\Delta}_1^{-1}\mathbf{K})^{-1}\mathbf{K}^{\mathrm{T}}\mathbf{\Delta}_1^{-1} & \mathbf{0} \\ \mathbf{0} & \mathbf{\Delta}_2^{-1}\mathbf{K}^{\mathrm{T}}(\mathbf{\Delta}_1 - \mathbf{K}\mathbf{\Delta}_2^{-1}\mathbf{K}^{\mathrm{T}})^{-1}\mathbf{K}\mathbf{\Delta}_2^{-1} \end{bmatrix} +$$
$$r^2 \begin{bmatrix} \mathbf{0} & -\mathbf{\Delta}_1^{-1}\mathbf{K}(\mathbf{\Delta}_2 - \mathbf{K}^{\mathrm{T}}\mathbf{\Delta}_1^{-1}\mathbf{K})^{-1} \\ -(\mathbf{\Delta}_2 - \mathbf{K}^{\mathrm{T}}\mathbf{\Delta}_1^{-1}\mathbf{K})^{-1}\mathbf{K}^{\mathrm{T}}\mathbf{\Delta}_1^{-1} & \mathbf{0} \end{bmatrix}. \quad \text{(S7)}$$

The Jacobian for the integration is

$$\mathbf{\Upsilon} = [\mathbf{\Upsilon}_1 \quad \mathbf{\Upsilon}_2], \quad \text{(S8)}$$

where

$$\mathbf{\Upsilon}_1 = [\tilde{\alpha}_1 a_1^{-1} \quad -\tilde{\alpha}_1 \tau_1] \quad \text{(S9)}$$

and

$$\mathbf{\Upsilon}_2 = [\tilde{\alpha}_2 a_2^{-1} \quad -\tilde{\alpha}_2 \tau_2] \quad \text{(S10)}$$

with $\tilde{\alpha}_1 = a_1\lambda_1^{-1}$, $\tilde{\alpha}_2 = a_2\lambda_2^{-1}$, $\tau_1 = \lambda_1^{-1}$, and $\tau_2 = \lambda_2^{-1}$.

The variance of the TIA becomes

$$V(\tilde{A}) = \mathbf{\Upsilon}\mathbf{\Sigma_P}\mathbf{\Upsilon}^{\mathrm{T}} = r^2\mathbf{\Upsilon}_1\Delta_1^{-1}\mathbf{\Upsilon}_1^{\mathrm{T}} + r^2\mathbf{\Upsilon}_2\Delta_2^{-1}\mathbf{\Upsilon}_2^{\mathrm{T}} +$$
$$r^2\mathbf{\Upsilon}_1\mathbf{\Delta}_1^{-1}\mathbf{K}(\mathbf{\Delta}_2 - \mathbf{K}^{\mathrm{T}}\mathbf{\Delta}_1^{-1}\mathbf{K})^{-1}\mathbf{K}^{\mathrm{T}}\mathbf{\Delta}_1^{-1}\mathbf{\Upsilon}_1^{\mathrm{T}} +$$
$$r^2\mathbf{\Upsilon}_2\mathbf{\Delta}_2^{-1}\mathbf{K}^{\mathrm{T}}(\mathbf{\Delta}_1 - \mathbf{K}\mathbf{\Delta}_2^{-1}\mathbf{K}^{\mathrm{T}})^{-1}\mathbf{K}\mathbf{\Delta}_2^{-1}\mathbf{\Upsilon}_2^{\mathrm{T}} -$$
$$r^2\mathbf{\Upsilon}_1\mathbf{\Delta}_1^{-1}\mathbf{K}(\mathbf{\Delta}_2 - \mathbf{K}^{\mathrm{T}}\mathbf{\Delta}_1^{-1}\mathbf{K})^{-1}\mathbf{\Upsilon}_2^{\mathrm{T}} -$$
$$r^2\mathbf{\Upsilon}_2(\mathbf{\Delta}_2 - \mathbf{K}^{\mathrm{T}}\mathbf{\Delta}_1^{-1}\mathbf{K})^{-1}\mathbf{K}^{\mathrm{T}}\mathbf{\Delta}_1^{-1}\mathbf{\Upsilon}_1^{\mathrm{T}}. \quad \text{(S11)}$$

Noting that $V(\tilde{A})$, and hence all constituent terms, are scalar in combination with $(\mathbf{\Delta}_2 - \mathbf{K}^{\mathrm{T}}\mathbf{\Delta}_1^{-1}\mathbf{K})^{-1}$being symmetric, $\mathbf{\Upsilon}_2(\mathbf{\Delta}_2 - \mathbf{K}^{\mathrm{T}}\mathbf{\Delta}_1^{-1}\mathbf{K})^{-1}\mathbf{K}^{\mathrm{T}}\mathbf{\Delta}_1^{-1}\mathbf{\Upsilon}_1^{\mathrm{T}} = \left[\mathbf{\Upsilon}_1\mathbf{\Delta}_1^{-1}\mathbf{K}(\mathbf{\Delta}_2 - \mathbf{K}^{\mathrm{T}}\mathbf{\Delta}_1^{-1}\mathbf{K})^{-1}\mathbf{\Upsilon}_2^{\mathrm{T}}\right]^{\mathrm{T}} = \mathbf{\Upsilon}_1\mathbf{\Delta}_1^{-1}\mathbf{K}(\mathbf{\Delta}_2 - \mathbf{K}^{\mathrm{T}}\mathbf{\Delta}_1^{-1}\mathbf{K})^{-1}\mathbf{\Upsilon}_2^{\mathrm{T}}$ and defining

$$D_1 = \mathbf{\Upsilon}_1\Delta_1^{-1}\mathbf{\Upsilon}_1^{\mathrm{T}}, \quad \text{(S12)}$$

$$D_2 = \mathbf{\Upsilon}_2\Delta_2^{-1}\mathbf{\Upsilon}_2^{\mathrm{T}}, \quad \text{(S13)}$$

$$C_1 = \mathbf{\Upsilon}_1 \mathbf{\Delta}_1^{-1} \mathbf{K} \left(\mathbf{\Delta}_2 - \mathbf{K}^{\mathrm{T}} \mathbf{\Delta}_1^{-1} \mathbf{K} \right)^{-1} \mathbf{K}^{\mathrm{T}} \mathbf{\Delta}_1^{-1} \mathbf{\Upsilon}_1^{\mathrm{T}}, \tag{S14}$$

$$C_2 = \mathbf{\Upsilon}_2 \mathbf{\Delta}_2^{-1} \mathbf{K}^{\mathrm{T}} \left(\mathbf{\Delta}_1 - \mathbf{K} \mathbf{\Delta}_2^{-1} \mathbf{K}^{\mathrm{T}} \right)^{-1} \mathbf{K} \mathbf{\Delta}_2^{-1} \mathbf{\Upsilon}_2^{\mathrm{T}}, \tag{S15}$$

and

$$\rho = \mathbf{\Upsilon}_1 \mathbf{\Delta}_1^{-1} \mathbf{K} \left(\mathbf{\Delta}_2 - \mathbf{K}^{\mathrm{T}} \mathbf{\Delta}_1^{-1} \mathbf{K} \right)^{-1} \mathbf{\Upsilon}_2^{\mathrm{T}} \tag{S16}$$

leads to

$$V(\tilde{A}) = r^2 (D_1 + D_2 + C_1 + C_2 - 2\rho), \tag{S17}$$

where, in analogy with the mono-exponential case,

$$D_1 = \tilde{\alpha}_1^2 \frac{1}{a_1^2 Q_1} \left[1 + \frac{(\bar{t}_1 - \tau_1)^2}{s_1^2} \right], \tag{S18}$$

and

$$D_2 = \tilde{\alpha}_2^2 \frac{1}{a_2^2 Q_2} \left[1 + \frac{(\bar{t}_2 - \tau_2)^2}{s_2^2} \right]. \tag{S19}$$

There are straight-forward interpretations of the different terms in (S17) with D_1 and D_2 representing the propagation of dispersion for the two phases individually; C_1 and C_2 represent corrections to D_1 and D_2; and ρ represent the correlation between the two phases.
